# Plant apomixis is rare in Himalayan high-alpine flora

**DOI:** 10.1038/s41598-019-50907-5

**Published:** 2019-10-07

**Authors:** Viktorie Brožová, Petr Koutecký, Jiří Doležal

**Affiliations:** 1 Department of Botany, Faculty of Science, University of South Bohemia, Branišovská 1760, 370 05 České Budějovice, Czech Republic; 2 Institute of Botany, The Czech Academy of Sciences, Dukelská 135, Třeboň, 379 01 Czech Republic; 3 Laboratory of Tree Ring Research, University of Arizona, 1215 E. Lowell Street, Tucson, 85721 Arizona, USA

**Keywords:** Plant reproduction, Plant ecology, Ecosystem ecology

## Abstract

Gametophytic apomixis is a way of asexual plant reproduction by seeds. It should be advantageous under stressful high altitude or latitude environment where short growing seasons, low temperatures, low pollinator activity or unstable weather may hamper sexual reproduction. However, this hypothesis remains largely untested. Here, we assess the reproductive mode in 257 species belonging to 45 families from the world’s broadest alpine belt (2800–6150 m) in NW Himalayas using flow cytometric seed screen. We found only 12 apomictic species, including several members of Poaceae (*Festuca*, *Poa* and *Stipa*), Rosaceae (*Potentilla*) and Ranunculaceae (*Halerpestes*, *Ranunculus*), which are families typical for high apomict frequency. However, several apomictic species were newly discovered, including the first known apomictic species from the family Biebersteiniaceae (*Biebersteinia odora*), and first apomicts from the genera *Stipa* (*Stipa splendens*) and *Halerpestes* (*Halerpestes lancifolia*). Apomicts showed no preference for higher elevations, even in these extreme Himalayan alpine habitats. Additional trait-based analyses revealed that apomicts differed from sexuals in comprising more rhizomatous graminoids and forbs, higher soil moisture demands, sharing the syndrome of dominant species with broad geographical and elevation ranges typical for the late-successional habitats. Apomicts differ from non-apomicts in greater ability of clonal propagation and preference for wetter, more productive habitats.

## Introduction

There is a general opinion that gametophytic apomixis, the mode of plant reproduction via seeds formed by asexual way from gametophytic tissue, is more frequent in cold environments of higher latitudes and altitudes^1–4^. Apomicts might be better colonizers than sexual species in arctic and alpine areas because of uniparental reproduction and ability to set up populations by single individuals^5,6^, thereby compensating for reduced fecundity resulting from pollen limitation in small and isolated populations^7^. However, the hypothesis of higher frequency of apomictic plants in cold environments has been rarely tested^8^, but when tested, the prominent role of apomixis is questioned^8–10^. Several studies on *Ranunculus kuepferi* found clear connection between higher elevation and apomixis, but simultaneously between apomixis and polyploidy. It seems that these genomic and environmental features are bound together – polyploids appear rather in colder climate of high elevation, and polyploids have tendency to reproduce by apomixis^11–13^. As understanding of this polyploid complex increased, also opposite relationship was proposed – polyploidy might be bound to apomixis^14,15^. Here we analyse to our knowledge the largest dataset of alpine plant species to assess whether the occurrence of apomictic plants increase along Himalayan elevational gradient, starting around 3000 m and ending at the absolute limit of vascular plants at 6000 m a.s.l.

There are several mutually non-exclusive explanations why the frequency of apomicts should increase upwards or polewards. (1) Cold areas may lack pollinators necessary for certain plants to reproduce sexually^16,17^; this, however, does not explain pseudogamy where pollination is still required for endosperm development^18^. (2) As there is no recombination during meiosis, apomixis keeps all successful gene combinations and hence suitable adaptations, which is advantageous in special but stable habitat (occurrence of so called fixed heterozygosity in apomicts is reviewed by Gornall^19^). (3) Pleistocene glaciations have increased the proportion of apomictic species by promoting hybridisation and afterwards polyploidization between previously isolated populations that came in contact within glacial refugia^20,21^; isolation of such hybrids in the newly deglaciated arcto-alpine areas followed^22–25^. (4) Species in cold areas have mainly disjunctive populations, therefore, according to the Baker´s law^26,27^ (the law formulated by Stebbins^28^), the autonomic or self-compatible species might be better able of colonising these areas.

Other predictions, supporting the affinity of apomicts to cold areas, have been more recently added to the original above-mentioned arguments defined by Stebbins^5^. For instance, apomixis may save energy otherwise used for expensive process of meiosis^24^., or, the second way of sparing energy is creating no pollen, which, however, is not universal, as pseudogamous plants need pollen to fertilise their endosperm^29^. Nevertheless, some of the abovementioned arguments are just theories, which can be easily challenged. Firstly, the Baker´s law is a generalized view on behaviour of an apomictic population based only on few examples of apomictic animal populations. Secondly, the ecological argument presupposing absence of pollinators was recently refuted as there are still effective pollinators occurring in cold arctic and alpine habitats^30,31^. Moreover, pollination does not depend on pollinators solely, as partial or full autogamy is widespread among angiosperms including apomicts^32–34^. Despite that, all these theoretical predispositions about higher occurrence of apomixis in extreme conditions have been considered as a fact for a long time^1^ without proper testing.

The problematic of higher frequency of apomictic plants in cold environments was not thoroughly studied until recently (but see the extensive research on *R. kuepferi*^11–15^). Gregor^35^ made a statistical analysis of frequency of apomicts in phytosociological relevés from Central Europe. The apomixis was highly positively correlated with altitude in his compilation. On the contrary, Hörandl *et al.*^8^ tried to verify the theory of higher frequency of apomicts in the European Alps by analysing 14 subnival species, all dependent on insect pollinators, to find out whether there is some effect of pollinator limitation^8^. From these species, only *Potentilla crantzii* (Crantz) Beck ex Fritsch, an apomictic plant described earlier by Smith^36^ was apomictic while other species showed seeds originated by sexual way, indicating that apomixis might be rather rare in high elevations. However, there are extreme high mountain areas where the relation between apomixis and elevation might be stronger. Himalayas provide extensive alpine environment potentially less favourable for insect pollinators compared to European mountains and therefore more favourable for formation of apomixis due to severe ecological constraints^37^.

There are several ways how to study the apomixis, most effective being a flow cytometric seed screening (FCSS^38^), which became popular in the last few decades^8,15,39^. FCSS measures DNA content in mature angiosperm seeds and infer the reproductive mode based on DNA content of the embryo and the endosperm without a need to know the actual genome sizes or ploidy levels^40,41^. Sexual angiosperm seed derived from reduced gametes and normal double fertilization has 2C embryo (formed from a 1C egg cell and a 1C sperm cell) and 3C endosperm (a 2C central nucleus of the embryonic sac plus a 1C sperm cell)^7,42^. Apomictic seeds have different ratios the embryo and the endosperm genome sizes^43^. The ratios 2/4, 2/5 or 2/6 are the most frequent under automonous endosperm development, pseudogamy with a reduced male gamete and an unreduced male gamete, respectively^41^. However, there are many other possibilities depending on the actual way of embryo and endosperm formation and ploidy levels of taxa involved (in cases when pollen- and egg- parents have different ploidy levels) but most of these combinations are reliably distinguished from sexual seeds^39,44,45^.

It has been widely assumed that apomixis is frequently associated with vegetative, clonal propagation. Vegetative reproduction, in which parental genets produce new ramets capable of independent growth and dispersal^46^, is commonly associated with the perennial life form, longevity, and occurrence in cold habitats in which sexual recruitment is often restricted. According to Barrett^47^, extensive vegetative growth by clonal rhizomes, stolones and root sprouts can disrupt the functioning of sexual polymorphisms; mutations reducing fertility may lead to sexual dysfunction and even the loss of sex in populations in which clonal propagation predominates. However, there have been few efforts documenting whether apomicts differ from sexuals in the adaptive strategies including vegetative propagation and other ecophysiological traits enabling plants to cope with harsh alpine conditions.

**Figure 1:**
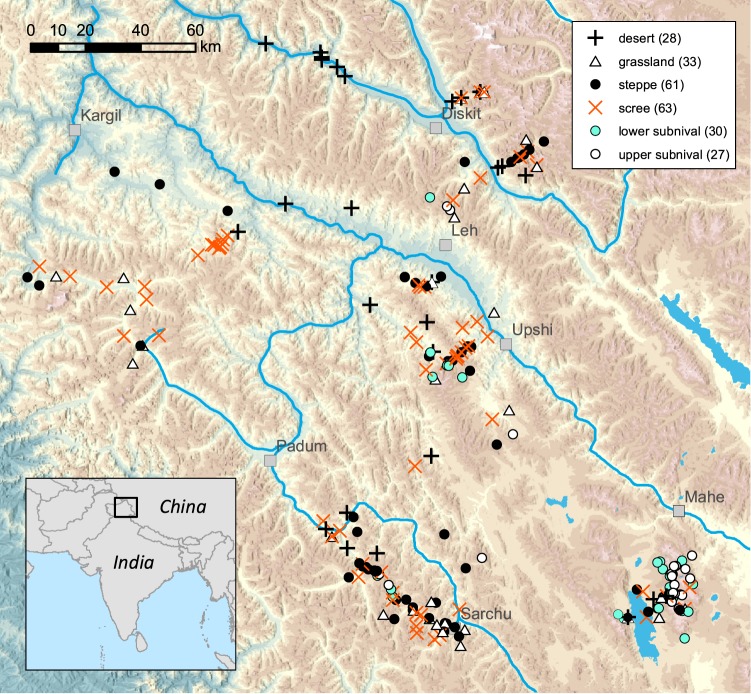
Map of localities in Ladakh. Background map is based on. OpenStreetMap data available under the Open Database License: http://opendatacommons.org/licenses/odbl/1.0/.

In this study, we focused on testing the hypothesis about increasing frequency of apomicts with increasing elevation in the northwestern Himalayas. By collecting 257 species along unprecedented elevation gradient including cold deserts, steppes, alpine meadows and subnival zone with world’s highest growing plants^37^ (Fig. 1), we aimed at (1) screening representative number of taxa across major habitats and the angiosperm groups for determining their reproductive mode using flow cytometry, (2) determining the specific way of reproduction in apomictic species, (3) linking the occurrence of apomictic species with habitat conditions, especially species’ elevational optima, (4) assessing whether apomicts differ from sexuals in their abilities to cope with harsh alpine conditions by comparing their plant ecophysiological traits, clonal growth strategies and species ecological preferences.

## Results and Discussion

Out of 257 measured taxa, reproductive mode was inferred for 220 while 37 species had no detectable endosperm signal. 211 species showed sexual way of seeds formation (sometimes sexuality was found together with apomixis in a species). We found only 13 apomictic taxa (including two subspecies of *Poa pratensis*) belonging to seven genera and four families. Most of the apomictic species studied in the Himalayas were not tested for apomixis yet, so we cannot compare reproduction systems within species with other locations. The most of the few cases, which we were able to compare across species ranges, indicate that apomictic species have the same reproduction system at lower elevations as well as at higher elevations

3 out of 12 apomictic species belong to the genus *Potentilla* (Rosaceae), namely *Potentilla pamirica* Th.Wolf, *Potentilla sericea* L., *Potentilla sojakii* Dikshit & Panigrahi and 4 belong to the genus *Poa* (Poaceae): *Poa alpina* L., *Poa attenuata* Trin., *Poa pratensis* L., *Poa sterilis* M.Bieb. Other apomictic species were *Biebersteinia odora* Fisch. (Biebersteiniaceae), *Festuca olgae* (Regel) Krivot., *Stipa splendens* Trin (=(*Achnatherum splendens* (Trin.) Nevski) (both Poaceae), *Halerpestes lancifolia* (Bertol.) Hand.-Mazz., and *Ranunculus membranaceus* Royle (both Ranunculaceae). *Poa, Potentilla*, and *Ranunculus* are genera well-known for high frequency of apomictic species. Although *Stipa splendens* have no apomictic relatives in the genus and apomicts in the genus *Festuca* are also rather rare, both genera belong to family Poaceae, which is among the three families with the highest frequency of apomixis (Asteraceae, Rosaceae, Poaceae). Although there is no information on apomixis in *Halerpestes*, this genus is closely related to *Ranunculus*^48^ in which apomixis in the family is known. There is no information on apomixis within Bierbersteiniaceae yet; our data on *B. odora* are the first for this family in this respect.

### Differences in plant traits and elevation optima between apomictic and sexually reproducing species

**Figure 2:**
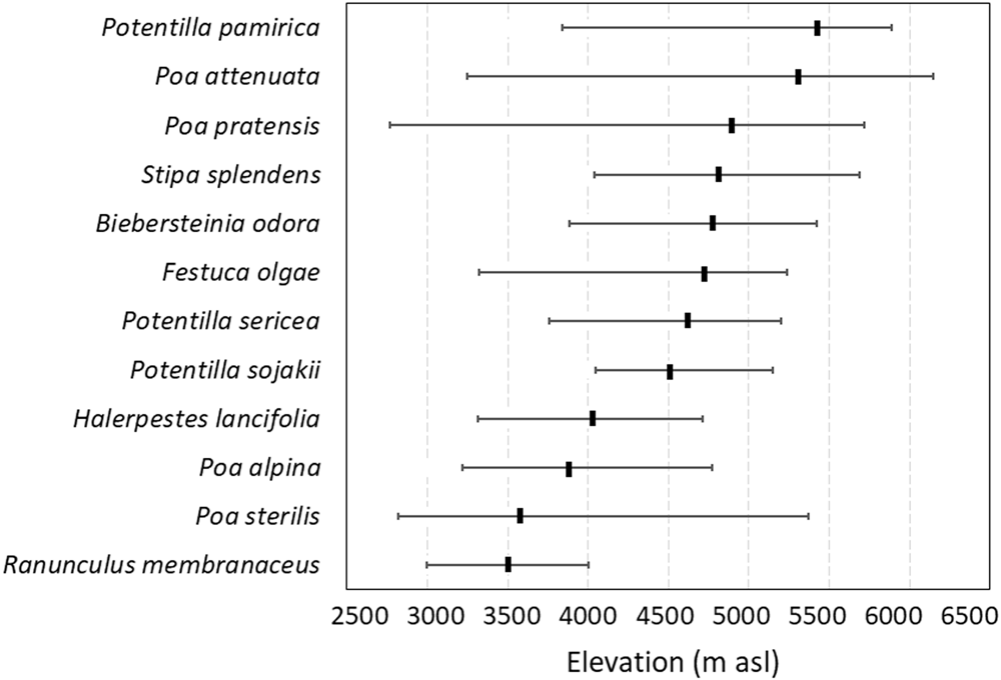
Apomictic species and their elevation optima and ranges in Ladakh, NW Himalayas. Note that it spans the whole range of elevations in the area and the apomictic species are not concentrated in higher elevations.

Apomicts and sexuals did not differ in elevation optima, minima and maxima, Raunkiær’s plant life-forms, leaf and root nutrient concentrations and root non-structural carbohydrates (Table 1). They however significantly differed in elevation ranges (pseudo F = 5.1, P = 0.035), clonal growth forms (pseudo F = 3.3, P = 0.004), ecological indicator values (pseudo F = 2.5, P = 0.031), and geographical occurrence (pseudo F = 12.7, P = 0.004). Apomictic species were clonal rhizomatous graminoids and forbs, with higher frequency of occurrence and soil moisture demands, sharing the syndrome of dominant, competitive species with broad geographical and elevation ranges (Fig. 2) typical for the stable, late-successional habitats. Apomicts did not show preference for higher elevations, even in these extreme Himalayan high-mountain habitats; they were instead bound to taxonomically related groups known for apomixis.

**Table 1:** List of plant functional traits and ecological indicator values measured on studied apomictic and non-apomictic species, their mean values and significant differences (ns, non-significant; *P < 0.05; **P < 0.01; ***P < 0.001).

Traits	Apomictis	Non-apomictis	P
Whole plant traits:			
Plant height (cm)	35.05	32.46	ns
Seed traits			
Embryo/endosperm ratio	2.49	1.49	***
Percentage of endosperm	32.03	32.17	ns
Embryo nuclei counts	1206.8	1777.1	*
Endosperm nuclei counts	550.43	678.34	ns
Organ traits:			
Leaf nitrogen content (%)	2.17	2.57	ns
Leaf phosphorus content (%)	0.16	0.18	ns
Leaf carbon content (%)	41.77	40.26	ns
Leaf d^13^carbon (‰)	−26.59	−26.81	ns
Leaf d^15^nitrogen (‰)	2.16	3.16	ns
Leaf dry matter content (mg/g)	341.9	293.9	ns
Root nitrogen content (%)	0.96	1.16	ns
Root phosphorus content (%)	0.12	0.13	ns
Starch content (%)	4.60	4.21	ns
Fructan content (%)	4.03	4.50	ns
Soluble sugars (%)	4.93	4.92	ns
Total nonstructural carbohydrates (%)	11.41	12.05	ns
Raunkiær’s plant life-forms			
Therophytes	0.00	0.10	ns
Geophytes	0.08	0.06	ns
Hemicryptophytes	0.92	0.68	ns
Chamaephytes	0.00	0.12	ns
Phanerophytes	0.00	0.05	ns
Clonal growth forms			
Clonal (%)	0.85	0.33	***
Hypogeogeic rhizomes <10 cm (%)	0.08	0.01	ns
Hypogeogeic rhizomes >10 cm (%)	0.15	0.07	ns
Epigeogeic rhizome <10 cm (%)	0.54	0.15	***
Epigeogeic rhizome >10 cm (%)	0.08	0.01	ns
Tap root, caudex and short increments (%)	0.15	0.38	ns
Tap root, caudex and long increments (%)	0.00	0.14	ns
Cushions with no advantitious buds on roots (%)	0.00	0.03	ns
Non-spreading integrators (%)	0.15	0.45	***
Spreading integrators (%)	0.00	0.13	ns
Non-spreading splitters (%)	0.62	0.24	***
Spreading splitters (%)	0.31	0.08	*
Species ecological information			
Landscape abundance (frequency)	1227	487	***
Elevation optima (m a.s.l)	4430	4347	ns
Elevation range (m)	2044	1677	*
Elevation minima (m)	3217	3364	ns
Elevation maxima (m)	5261	5041	ns
Indicator value_Stability	1.69	1.88	ns
Indicator value_Moisture	2.15	1.74	*
Indicator value_Salinity	0.15	0.24	ns
Indicator value_Nutrient	1.92	1.70	ns
Indicator value_Shade	2.54	2.76	ns

Our findings corroborate previous studies^46^ indicating that apomixis is associated with the on-spot persistence, high plant longevities, and occurrence in stable habitats where extensive clonal growth may disrupt the functioning of sexual polymorphisms, resulting in mutations reducing fertility, which may lead to sexual dysfunction and even the loss of sex. Apomicts in NW Himalayas form ecologically distinct group differing from non-apomicts in greater ability of clonal propagation and preference for wetter, more productive habitats. This implies that apomicts are often effective in reproducing and spreading, but successful are only when growing in rather stable habitats, being less successful in colonizing rapidly changing environment such as new substrate after deglaciation^49,50^.

### Apomictic species of genus *Potentilla*

**Figure 3:**
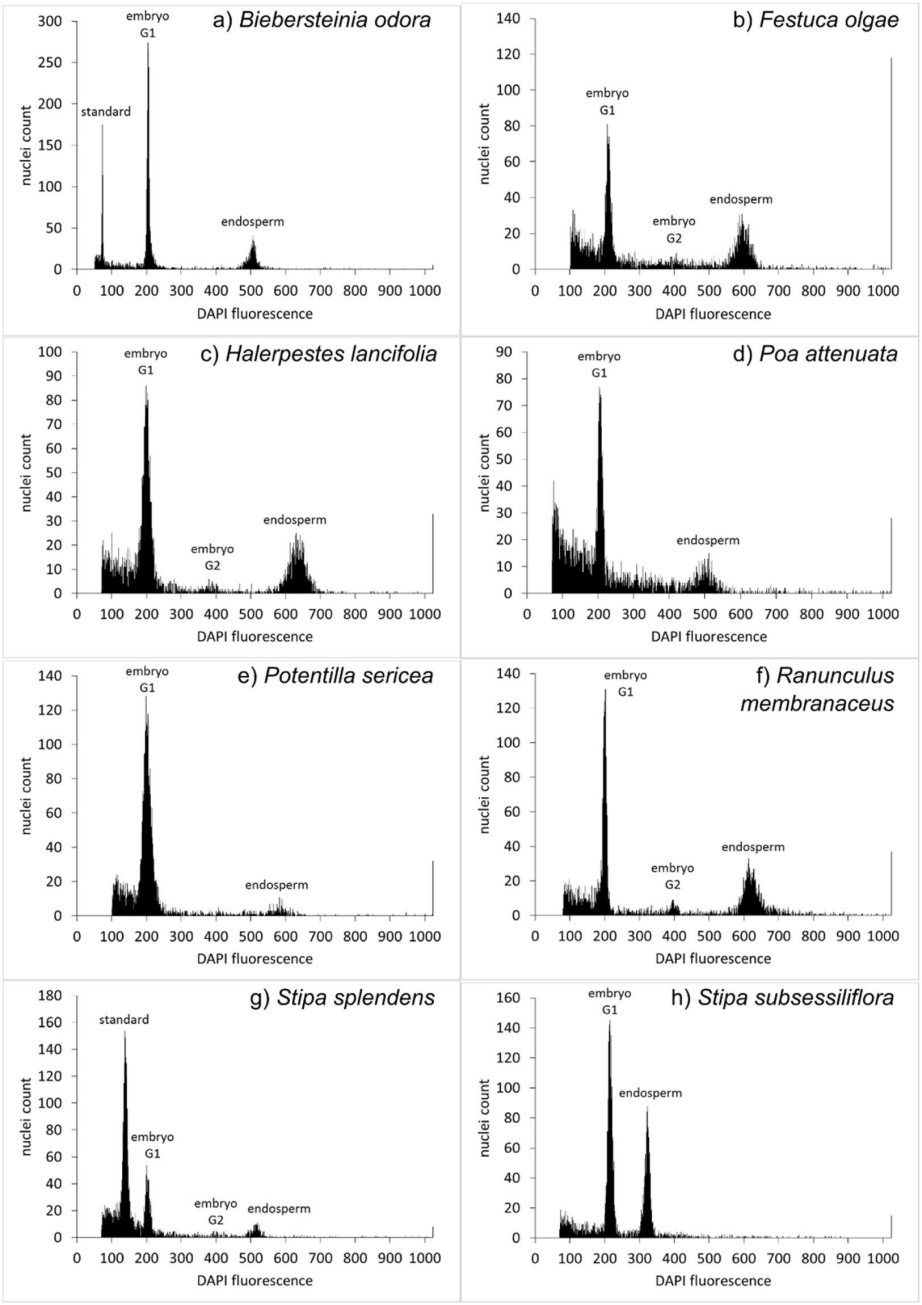
FCSS histograms of selected apomictic species and a sexual species *Stipa subsessiliflora*. Peeks show the amount of nuclei with relative DNA amount represented by intensity of DAPI fluorescence.

Three apomicts recruited from the genus *Potentilla*, including *P. pamirica*, *P. sericea*, and *P. sojakii*. They all showed pseudogamous development of seeds. The ratio of embryo/endosperm was mostly 2/6, which means fertilisation of central cell by an unreduced sperm cell or by two reduced sperm cells (Fig. 3e). The second possible way is more probable because we know from former studies that pollen of *Potentilla* is mostly reduced^51,52^. Moreover, this type of endosperm fertilisation was also observed in Rosaceae^45,53^. Alternatively, other species of higher different ploidy level might contribute unreduced-like pollen at site where several species co-occur^54^.

Apomixis in the genus has been well studied. Dobeš *et al.*^55^ provides a summary of reproduction systems for 22 series of the genus *Potentilla*, of which apomixis was confirmed in 14 series, but the sexuality co-occurred in 10 out of these 14 series. The apomictic series occur exclusively in phylogenetically young core *Potentilla*. However, study of apomixis in this genus has its limits. The first is caused by hardly detectable endosperm tissue^56,57^, which is sometimes completely missing^58^. In our case, 44 out of 57 samples were without detectable endosperm tissue. The second obstacle arises with occasional deviation from standard eight nuclei in embryo-sac. Dobeš *et al.*^55^ observed only one polar nucleus in endosperm of *Potentilla indica*, with flow cytometer histograms indicating regular sexuality; however, an AFLP analysis of progeny revealed an apomictic origin of seeds.

In spite of missing data on reproduction mode in some Himalayan *Potentilla* spp., for some species within our dataset, reports from other areas are available. Predisposition for apomixis was already known in one species (*P. sericea*; polyploidization by hybridization)^59^. *Potentilla venusta* is known to be apomictic^55^; our analyses were, however, without readible signal and we therefore cannot determine the reproduction system. *Potentilla multifida* showed sexual reproduction in our sample, even at 5000 m a. s. l., while Dobeš *et al.*^55^ described 18 apomictic embryo-sacs, seven parthenogenetic embryos, and no seeds of sexual origin. The same case is *Potentilla atrosanguinea* with 15 out of 22 studied seeds being clearly apomictic in Dobeš *et al.*^55^, while all our samples were all sexual. Christoff & Papasova^60^ also described *P. atrosanguinea* as a sexual, but his material came from botanical gardens, and so it is not directly comparable with our wild populations. Concerning reproduction modes of other sampled species which were unfortunately without clear signal, Rani *et al.*^61^ observed meiotic abnormality in *Potentilla gelida* in Western Himalayas, while another study by Jeelani *et al.*^59^ showed polyploidy in *Potentilla sericea* but no meiotic abnormalities. There are no previous studies concerning breeding systems in *P. bifurca*, *P. evestita*, *P. pamirica*, *P. sojakii* or *P. turczaninowiana*. However, *P. bifurca* was involved in a phylogenetic study by Eriksson *et al.*^62^. It placed the species outside the core *Potentilla*, which suggests that *P. bifurca* should not be able to reproduce by apomixis, which was confirmed by our findings.

### Apomictic species in Ranunculaceae

The species *Ranunculus membranaceus* and closely related species *Halerpestes lancifolia* produced seeds in the analogous way as *Potentilla* mentioned above: the ratio was always 2/6, which indicates pseudogamy with one or two sperm cells involved in formation of endosperm (Fig. 3c,f). This type of endosperm fertilisation was described in *Ranunculus auricomus*^7,45,63^, the intensively studied facultative apomictic complex^64^. We can expect that apomixis in *R. membranaceus* is of the same type, as this species is sister to the clade containing the *R. auricomus* complex^65^. As apomictic *Ranunculus* spp. are extensively investigated, mechanism of polyploid apomictic seeds has been suggested^13^. It seems that polyploidization happens regularly mainly via triploid bridge with an unreduced egg cell. This loss of meiosis in female gametes might be the first step for development of apomixis in *Ranunculus* polyploids. However, Klatt *et al*.^15^ supported rather stress-dependent loss of sex resulting in polyploidization rather than polyploidization as a preadaptation for apomixis. The second apomictic species of Ranunculaceae was *Halerpestes lancifolia* with the same embryo/endosperm ratio as *R. membranaceus*. It is an unexpected observation, because no other apomictic genera except *Ranunculus* were discovered within the family Ranunculaceae so far. Nevertheless, the genus *Halerpestes* is closely related to *Ranunculus* s.str.^66^ and occurrence of the apomixis of *Ranunculus*-type might be expected.

### Apomictic species in Poaceae

The family Poaceae is also known for high frequency of apomictic species. In our data set, 16 species of Poaceae were tested, from which one is an apomictic species of the genus *Festuca* (namely *Festuca olgae*), four belong to the genus *Poa* (*Poa alpina*, *Poa attenuata, Poa pratensis, Poa sterilis*) and one species belong to the genus *Stipa* (*Stipa splendens*).

*Festuca olgae* is a facultative type of an apomict. We measured 22 seeds from 3 plants and just two seeds were apomictic with endosperm/embryo ratio 6/2 (Fig. 3b). Such facultative apomixis is known to occur in Poaceae^67^ and it happens mostly in an aposporous plant which creates an embryo-sac directly from nucellus^68^. A facultative apomict might have an advantage over strict sexual or apomictic. In fact, it has advantages from both of them. The plant can produce genetically variable offspring to improve its features in changing environment, on the other hand it produces also stable offspring which is well adapt to current conditions. Even though apomixis in the genus is not very common, it was observed already^69^ and; in addition, the genus *Festuca* is quite close related to the genus *Poa*^70^, which is one of the best known apomictic genera^67^.

The genus *Poa* had mostly pseudogamous mode of reproduction in our analyses (in majority cases with the ratio 5/2, in the only one case with the ratio 6/2; Fig. 3d). This type of apomixis appeared in all four tested species: *P. alpina*, *P. attenuata*, *P. pratensis* and *P. sterilis*. One seed of *Poa attenuata* from our measurement shows autonomy. Although the presence of pseudogamy and autonomy in one species is very rare, it has been described in *Poa nervosa*^67^. Nevertheless, the second and more probable explanation can be found: Nogler^63^ described a situation where one of two central nuclei was degraded, therefore one unreduced or two reduced sperm cells may cause the “autonomic” ratio.

Similarly to *Potentilla* spp., the genus *Poa* was studied well for apomixis and for many species data about reproduction mode are available. *Poa attenuata* (collected on the north side of Ťan-Šan, in 1200 m a. s. l.) was tested by Kelley *et al.*^67^ with exclusively pseudogamous apomixis (with the embryo/endosperm ratio 2/5). Nevertheless, the genus is characteristic for facultative apomixis, which was demonstrated by two sexual seeds in two Himalayan *Poa* species (*P. pratensis subsp. staintonii*, *P. sterilis*). This phenomenon was described for both species^67^.

The case of *Stipa splendens* is also unique. There is no reported apomixis in *Stipa*^71^ or related taxa, however, our samples showed pseudogamous and autonomous endosperm formation, similarly to e.g. what was observed by Kelley *et al*.^67^ in *Poa nervosa*. Within the pseudogamous seeds, majority of *S. splendens* seeds had ratio 2/5 (Fig. 3g), but also the ratio 2/6 was not unique. One seed had a ratio 3.32 which should be formally closer to the ratio 2/7, nevertheless, this minor deviation is probably caused by pollen with bigger genome size. That at least two genome sizes of embryos occur in a population showed our measurements with standard (Supplementary Table S1), corresponding probably to diploids and triploids, so the ratio close to 2/7 on a diploid mother may correspond to pollination with triploid pollen. Our data revealed also two samples of *Stipa splendens* with unusual peaks ratios 2.11 and 2.17. In one case the sample might be a triploid and the ratio was than consistent with pollination with diploid’s reduced (haploid) pollen (3/7), in the other case we do not have a clear explanation for the observed pattern (the role of rare embryonic sac modifications and/or unbalanced pollen may be suspected).

### Apomixis in *Biebersteinia*

*Biebersteinia odora* was one of the unexpected apomictic species revealed in Ladakh as there is no record of apomixis in the family so far. Moreover, more ploidy levels of embryos and several ways of endosperm origin were revealed. The results showed stable genome size ratio of mother plant to standard (2.7–3), however, the ratio of embryo to standard varied (Supplementary Table S2).

In two populations of three sampled populations, genome size of maternal plants (measured from leaves) was uniform, and these plants produced mainly embryos of the same genome size; we consider them diploid (normal). Majority of seeds showed the embryo/endosperm ratio 2/5 (Fig. 3a), which indicates pseudogamous apomictic embryonic sac pollinated by one sperm of reduced pollen. Indeed, it is known that in apomictic groups mainly female meiosis is hampered while male meiosis often remains unchanged^71^. About 10% of seeds showed the ratios 2/6 (diploid pollen or two sperms of haploid pollen) or 2/7 (tri-nucleate central cell and haploid pollen). Single seeds had the profiles 2/8 (tri-nucleate central cell and one diploid or two haploid sperms), 2/10 (probably tri-nucleate central cell and two diploid sperms), we also found a few seeds with ratios indicating presence of aneuploid (between 1x and 2x pollen). One seed had 4x embryo and 6x endosperm and most likely is so called BIII hybrid (firstly described by firstly Rutishauer^72^, also “U-hybrids” by Asker^73^) resulting from fertilization of apomictic (unreduced) embryonic sac with diploid pollen. Such embryos are usually formed as 2n + n hybrids and, more rarely, as 2n + 2n hybrids^74^, and are important mechanism allowing ploidy level change. One seed had probably 3/8 profile; we do not have any simple explanation of its origin (types or reproduction systems are showed in the Supplementary Table S2). The last population showed remarkable variation in embryo genome size/ploidy levels. Unfortunately, it was the first population of *B. odorata* we collected and as we did not expect any variation, leaf samples were not available. Most of the embryos had calculated ploidy levels between 2x and 3x, some 2x and some 4x. About half of seeds had similar embryo/endosperm ratio as above (2/5 to 2/8) while the rest are probably aneuploid. Altogether, the recorded variation in all populations show that there are probably more ploidy levels in *B. odora* populations and probably a mixture of sexual and apomictic processes, involving also unbalanced gametes of odd ploidy levels (probably triploids). However, to understand this variation, sampling of more populations, more individuals from each population and both maternal plant and seed ploidy levels is needed. *Biesternia odorata* is thus an interesting organism for further study.

### Absence of apomixis in certain families

Finally, very important is to mention the families which we expected to be apomictic but no apomictic representative was found. Firstly, Asteraceae contains considerable amount of all known apomictic species but we did not find any apomicts among 36 different species from several genera we tested. Among the tested genera there were for example *Crepis*^75^, *Leontopodium*^76,77^, *Erigeron*^78–82^ which all possess apomixis but our samples were clearly sexual. We analysed many species of *Artemisia*^83,84^ which is known for apomictic tendencies but with the same negative result. On the other hand we did not have an opportunity to sample *Taraxacum*; it is likely that in this strongly apomictic genus^85^ some apomictic lineages might be present even in the Ladakh flora. Among Boraginaceae with known apomixis, the genus *Cynoglossum*^86^ in our dataset was sexual. Similarly it holds true for the other families with sporadic presence of apomixis (e.g. Brassicaceae^87,88^ or Lamiaceae^89^).

## Conclusion

Data obtained from seed samples collected in Ladakh revealed 12 species with gametophytic apomixis out of total 257 species collected. Among 12 apomictic species, four species were already described as apomictic (*Poa alpina, Poa attenuata, Poa pratensis, Poa sterilis*). Most of the apomictic species were not investigated through apomixis yet, therefore we cannot say whether there is some diversity between reproduction systems in different geographical conditions within the species. Nevertheless, in genera *Potentilla*, *Poa* and *Ranunculus* are well-known apomicts and apomixis within these genera was expected. Newly discovered apomixis in *Halerpestes lancifolia*, *Stipa splendens* and *Biebersteinia odora* was surprising and this finding is a valuable contribution to knowledge about apomictic groups. Our results clearly show that Ladakh apomicts mostly occur in taxonomical groups or lineages with already known apomictic species, which leads to negation of the theory of higher incidence of apomicts in high mountains and can reflect only tendency to apomixis within individual taxonomical lineages. Furthermore, in Ladakh occur two sexual species of the genus *Potentilla*, which were already described as possible apomictic. The trait-based comparison revealed that apomicts differed from sexuals in comprising more rhizomatous graminoids and forbs, higher soil moisture demands, sharing the syndrome of dominant species with broad geographical and elevation ranges typical for the late-successional habitats. Apomicts in NW Himalayas have greater ability of clonal propagation and preference for wetter, more productive and stable habitats where they effectively reproduce and spread; they seems to be less successful as pioneers in climate-induced uphill migration or new substrate colonization after deglaciation.

To sum up, predisposition for apomixis seems to be bound to specific phylogenetic lineages. The genetic principles of development of apomixis are still not fully described, but it is known that it includes many genetic and epigenetic mechanisms^90^. As the mechanism is so complex, apomixis is not a dominant reproductive strategy (less than 1% of Angiosperms^91^) and its establishment is difficult and is thus known only in a few lineages which succeeded in evolving such unusual method of asexual dispersal.

## Material and Methods

### Study area

The study was conducted in the Himalayan Mts. in Ladakh, Jammu and Kashmir State, India. Ladakh encompasses ca. 80.000 km^2^ of the Trans-Himalaya, delimited by the Eastern Karakoram Range in the north, the Great Himalaya Range in the southwest and Tibetan Plateau in the east. The studied localities ranged from 3000 m in Indus-Shayok-Nubra Valleys to 6150 m in Tso Moriri Lake area (Fig. 1). Desert and semi-desert occupy the (relatively) lowest elevations of the Indus Valley and its major tributaries (ca. 2900–3800 m a.s.l.). The subalpine vegetation belt stretches from approximately 3800 up to 5000 m a.s.l. in east Ladakh, and is widely dominated by cold steppe vegetation^37^. The alpine belt extends between ca. 4500 and 5200 m (occasionally to 5500 m) a.s.l. and hosts alpine grasslands, including the characteristic moist alpine turf of *Kobresia pygmaea* (C.B.Clarke) C.B.Clarke. A very sparse subnival vegetation zone is developed up to 6150 m a.s.l.

The elevational gradient in our study region is tightly related to a temperature and aridity gradient^49,50^. It is therefore warmer at low-elevation deserts, with intense drought due to lower precipitation and colder and more humid at high-elevation alpine and subnival zones. The length of the growing season decreases linearly with increasing elevation, from 270 to 30 frost-free days, while mean growing season temperature decreases from 14 to 3 °C between 3000 and 6150 m. Daily temperatures in the study area vary from 0 to 30 °C in summer and from –40 to –10 °C in winter. Annual precipitation is about 50 mm in the desert and semi-desert belt (falling mostly during the Indian summer monsoon), 100 mm in the alpine steppes, and 150–250 mm in the alpine and subnival zones^92^. Precipitation above 5000 m a.s.l. is mostly falling as snow and is more frequent in summer than in winter^37^. Soils have a coarse-grained structure, with a high percentage of large gravel, low water and organic matter content, high pH (7–8) and relatively high concentrations of total N and P^93^.

### Seed collections

Seeds were collected during August–September 2013–2015 at 137 localities. To reveal possible variability in reproduction systems, up to five individuals per species and population were collected; in selected species 10–15 populations was sampled. Seeds from each individual were stored in a separate bag. We used also some seeds from 25 species collected in 2009 (for a different study), however, in that case only population samples containing a mixture of seeds from several individuals were available. The seeds were stored in paper bags, allowing air to flow into the bag drying the seeds. Vouchers are deposited in herbarium of Institute of Botany, Academy of Science of the Czech Republic, Třeboň. Seeds of 36 species were collected directly from the herbarium vouchers that were collected between years 1999–2006. Locality details for all samples are specified in Supplementary Table S3.

### Flow cytometry

The relative fluorescence intensities of embryo and endosperm nuclei were analysed using the Partec PA II flow cytometer (Partec GmbH., Münster, Germany, now Sysmex) equipped with a mercury arch lamp. DAPI was used as a DNA stain. At least 5,000 particles were recorded. Two different protocols of sample preparation were tested and the one that gave sufficient results for a particular species (low coefficients of variance of the peaks and low background noise) was selected. (1) One-step protocol employing a seed buffer (Matzk *et al.*^38^ with minor modifications of Krahulcová & Suda^94^). Seeds were chopped with a razor blade in 1 ml of the seed buffer [0.1 M Tris, 5 mM MgCl_2_.6H_2_O, 85 mM NaCl, 0.1% (v/v) Triton X-100]. The suspension with filtrated through a 42 μm mesh and DAPI was added immediately after filtration (final concentration 4 μl/ml). The samples were analysed after a few minutes of staining. (2) Simplified two step protocol (Doležel *et al.*^95^, with minor modifications). Seeds were chopped in 400 μl of ice-cold Otto I buffer [0.1 M citric acid monohydrate, 0.5% (v/v) Tween 20]. The suspension with filtrated through a 42 μm mesh. After several minutes of incubation at room temperature, 800 μl of the staining solution was added, consisting of Otto II buffer [0.4 M Na_2_HPO_4_.12H_2_O], β-mercaptoethanol (0.2%) and DAPI (final concentration 4 µl/ml). Samples were run on the flow cytometer after several minutes of staining. In case of an insufficient results, different incubation time (both longer and shorter) were tested.

One to three seeds originating from one individual were pooled in a sample (however, in case of insufficient quality one-seeded analysis was always performed). If available, at least 3 seeds per individual and at least 3 individuals per population were analysed. In case of very small seeds, five or even ten seeds per sample were pooled to get strong enough signal. When genome or ploidy variation or variation in reproductive mode was suspected from pooled samples, additional analyses of single seeds were performed. In cases of genome size variation (in *Stipa splendens* Trin. and *Biebersteinia odora* Stephan ex Fisch.), samples were measured with an internal standard, which was a leaf tissue of *Pisum sativum* ‘Ctirad’ (2C = 9.09^96^) or of *Bellis perennis* (2C = 3.38 pg). For *Biebersteinia odora*, during the second fieldwork in 2014 we collected also leaf samples of the individuals from which seeds were collected. The leaf samples were dried in silica-gel and analysed using the two-step protocol (see above). Number of measured seeds and the protocol used are specified in Supplementary Table S4 for each species.

The fluorescence histograms were analysed in FlowJo 10 software (FlowJo LLC, Ashland, Oregon). The fluorescence intensity, coefficient of variation and number of nuclei were recorded for the G_0_/G_1_ peaks of the embryo and the endosperm. In case when the first peak of the endosperm was low due to strong endopolylpoidy (e.g. in *Astragalus* spp*., Oxytropis* spp*., Cicer arietinum* L.*, Colutea nepalnesis* Sims*, Atriplex schugnanica* Iljin*, Corispermum tibeticum* Iljin*, Christolea crassifolia* Cambess*, Galium pauciflorum* Willd. ex DC., *Leptorhabdos parviflora* (Benth.) Benth*., Pedicularis* spp.), the parameters of the second peak of the endosperm were recorded and the mean fluorescence intensity divided by the two. The ratio of the mean fluorescence intensities of the embryo and the endosperm was calculated and a reproductive system was inferred from it following Matzk^41^.

For variable *Biebersteinia odora* seeds measured with the internal standard, we calculated the relative genome sizes of the embryo and the endosperm to estimate their origin (genome sizes of the gametes and various modes of fertilization, following e.g. Dobeš *et al.*^39^). We considered sexual reproduction, apomixis with autonomous endosperm and apomixis with pseudogamy, i.e. fertilization of endosperm only with one or two reduced or unreduced sperms. Among the different scenarios, the one under which all calculated gamete genome sizes are close to the embryo genome size (unreduced gametes) or half of it (reduced) was considered the most likely. In a few cases calculated relative genome sizes were multiples of the embryo genome size, indicating that pollen probably originated from an individual of higher ploidy level.

### Relative size of embryo and endosperm in seeds

Furthermore, we prepared a summary of a relative amount of the endosperm in seeds, based on counts of nuclei of the embryo and the endosperm peaks (Supplementary Table S5). Percentage of endosperm in a seed was expressed as the ratio of count of nuclei of the endosperm peak to the sum of both peaks. An average of percentage of endosperm in a seed was counted for each family. This summary might serve as a tool for following studies dealing with FCSS, including identifying of families without detectable endosperm.

### Plant morphological and ecophysiological trait measurement

To assess which traits are associated with apomicts and those reproducing sexually, several plant traits, clonal growth strategies and species ecological information for all 257 species analysed for the mode of reproduction were recorded based on Cornelissen *et al.*^97^ and Doležal *et al.*^37,98^. More than 10 individuals were collected for each species at different elevations covering most of the species altitudinal ranges in Ladakh during several expeditions in years 2008–2016^37^. We measured several plant traits relevant to competitive ability (plant height), allocation (total, leaf, stem and root biomass), growth (SLA - specific leaf area, LDMC and StDMC - leaf and stem dry matter content, leaf and root NP concentrations), carbon storage (starch, fructans as non-structural carbohydrates), drought and frost tolerance (free sugars and sugar alcohols), water use efficiency (δ^13^C), generative (seed mass) and vegetative (clonality and lateral spread) propagation. These traits are important indicators of plant resource-use strategy, reflecting a fundamental trade-off between the efficient conservation of nutrients (low SLA, less negative δ^13^C, low foliar N and high LDMC) and the rapid production of biomass (e.g. high SLA, high foliar NP, low LDMC, long rhizomes). SHIMADZU UV - 1650PC spectrophotometer was used to determine phosphorus after digestion in HClO4. The δ^13^C, as well as total carbon and nitrogen, were measured using an elemental analyzer coupled to an IRMS at the Stable Isotope Facility, UC Davis, USA. Megazyme total starch assay procedure (www.megazyme.com) was used to determine starch and fructans; ethanol-soluble sugars were determined through anion exchange chromatography with pulsed amperometric detection. Soluble sugars included sugar alcohols such as glycerol, xylitol and arabitol, and simple sugars such as glucose, fructose, sucrose and galactose. The total non-structural carbohydrates (NSC) were calculated as the sum of all analysed carbohydrates.

### Vegetative clonal propagation

In addition to quantitative traits, each species was classified into one of 20 clonal growth forms based on which organ (rhizomes versus primary tap-roots) provides connections between offspring shoots, if this organ is short or long, if a plant is able to form adventitious roots, and if there are special storage organs. There are three exceptions: annuals and biennials, woody plants and cushion plants are assessed according to their whole morphology. Furthermore, species were divided into four space occupancy strategies, based on the rate of lateral spread (spreading–more than 10 cm per year; non-spreading–less than 10 cm per year) and persistence of connections between ramets (splitters–plants producing adventitious roots with main root decaying; integrators–plants not producing adventitious roots and/or with perennial main root^46^).

### Species elevational optima and ecological indicator values

To obtain a robust estimate of the elevational optima and ranges of the species, we calculated response curves fitted with HOF models^50^. Species response curves were derived from 4,150 vegetation plots (each 100 m × 100 m) sampled over the entire Ladakh between 1999 and 2014. The dataset contains more than 122,000 records of occurrence of vascular plant species along exceptional elevational gradient from 2800 m to 6150 m. Species’ optima on five environmental gradients (ecological indicator values) were derived from vegetation composition of 369 plots (each 100 m^2^) sampled in a stratified design to cover major vegetation types over the study area.

### Statistical analyses

To determine which set of traits best explain differences between apomicts and sexuals, we used multivariate RDA analysis using the CANOCO 5 software^99^. We first analysed all traits together, followed by analyses specific for each group of traits (whole plant traits, organ traits, clonal growth forms and species ecological information, see Table 1). Statistical tests were based on 9999 Monte Carlo permutations.

## Supplementary information

Supplementary Info
