# Supplementary material for: Plant apomixis is rare in Himalayan high-alpine flora
Source: Sci Rep. 2019 Oct 7;9:14386. doi: 10.1038/s41598-019-50907-5 (PMC6779868; doi:10.1038/s41598-019-50907-5)
Supplement: Supplementary file 1 — Supplementary Info [file 41598_2019_50907_MOESM1_ESM.pdf]

**Plant apomixis is rare in Himalayan high-alpine flora**

**Viktorie Brožová, Petr Koutecký, Jiří Doležal**

**Supplementary Table S1**: Results of measuring of *Stipa splendens* . Information about number of seeds in samples and about used protocol is written (O – two-step protocol using Otto buffers, M – one step protocol using seed buffer). Ratio of embryo to standard (eventually fist embryo to second embryo) is in the next columns (bright-blue – lower genome size, bright-red – higher genome size). Ratios of endosperm to embryo are noted and finally way of reproduction is described (green – pseudogamy, blue – autonomy). The sample 10 had undetectable endosperm.

| sample no. | number of seeds in a sample | FCSS protocol | embryo/standard | endosperm/embryo | endosperm 2/embryo | way of reproduction |
|---|---|---|---|---|---|---|
| 1 | 3 | M | × | 2.46 | 2.96 | pseudogamy, one reduced sperm cell / one unreduced sperm cell or two reduced sperm cells |
| 2 | 1 | M | 1.14 | 2.17 | | autonomy |
| 3 | 1 | M | 1.18 | 2.52 | | pseudogamy, one reduced sperm cell |
| 4 | 1 | M | 1.54 | 2.95 | | pseudogamy, one unreduced sperm cell or two reduced sperm cells |
| 5 | 1 | M | 1.46 | 2.54 | | pseudogamy, one reduced sperm cell |
| 6 | 1 | M | 1.12 | 2.6 | | pseudogamy, one reduced sperm cell |
| 7 | 1 | M | × | 2.49 | | pseudogamy, one reduced sperm cell |
| 8 | 1 | M | × | 2.11 | | autonomy |
| 9 | 1 | M | 1.2 | ? | | × |
| 10 | 1 | M | 1 | 3.32 | | pseudogamy, one unreduced sperm cell or two reduced sperm cells |
| 11 | 1 | M | 1 | 2.56 | | pseudogamy, one reduced sperm cell |

**Supplementary Table S2**: Measurements of *Biebersteinia odora* . In first column is number of individual. In the next columns are calculations of ratios of seed tissues and maternal genome size to standard. In the last column is written what is the most possible way of reproduction of the measured seed (green – pseudogamy, blue – autonomy, yellow – sexuality, orange – uncertain).

| individual | embryo/standard | endosperm/standard | endosperm/embryo | maternal plant/standard | way of reproduction |
|---|---|---|---|---|---|
| 1 | 3.117 | 12.112 | 3.886 | × | pseudogamy, endosperm with two unreduced sperm cells |
| 1 | 2.849 | 9.733 | 3.417 | | pseudogamy, endosperm with two unreduced sperm cells |
| 1 | 2.844 | 6.995 | 2.46 | | pseudogamy, endosperm with one reduced sperm cell |
| 1 | 2.865 | 11.232 | 3.921 | | pseudogamy, endosperm with two unreduced sperm cells |
| 1 | 2.817 | 8.396 | 2.981 | | pseudogamy, endosperm with one unreduced or two reduced cells |
| 2 | 2.857 | 11.257 | 3.94 | × | pseudogamy, endosperm with two unreduced sperm cells |
| 2 | 2.834 | 11.113 | 3.921 | | pseudogamy, endosperm with two unreduced sperm cells |
| 2 | 3.698 | 10.227 | 2.765 | | pseudogamy, endosperm with one unreduced or two reduced cells, triploid embryo |
| 2 | 3.121 | 10.013 | 3.209 | | pseudogamy, endosperm with one unreduced or two reduced cells, triploid embryo |
| 2 | 3.685 | 10.801 | 2.931 | | pseudogamy, endosperm with one unreduced or two reduced cells, triploid embryo |
| 3 | 3.981 | 9.812 | 2.465 | × | pseudogamy, endosperm with one reduced sperm cell, triploid embryo |
| 3 | 3.45 | 9.704 | 2.813 | | pseudogamy, endosperm with one unreduced or two reduced cells, triploid embryo |
| 3 | 3.696 | 9.293 | 2.515 | | pseudogamy, endosperm with one reduced sperm cell, triploid embryo |
| 3 | 5.613 | 11.557 | 2.059 | | autonomous apomixis, tetraploid embryo |
| 3 | 5.52 | 11.453 | 2.075 | | autonomous apomixis, tetraploid embryo |
| 3 | 2.801 | 9.637 | 3.44 | | pseudogamy, endosperm with one reduced sperm cell |
| 4 | 2.813 | 6.791 | 2.414 | 2.792 | pseudogamy, endosperm with one reduced sperm cell |
| 4 | 2.808 | 6.719 | 2.393 | | pseudogamy, endosperm with one reduced sperm cell |
| 4 | 2.781 | 6.895 | 2.48 | | pseudogamy, endosperm with one reduced sperm cell |
| 4 | 2.795 | 6.894 | 2.467 | | pseudogamy, endosperm with one reduced sperm cell |
| 4 | 2.825 | 6.952 | 2.461 | | pseudogamy, endosperm with one reduced sperm cell |
| 4 | 2.824 | 6.868 | 2.432 | | pseudogamy, endosperm with one reduced sperm cell |
| 4 | 2.779 | 8.165 | 2.939 | | pseudogamy, endosperm with one unreduced or two reduced cells |
| 5 | 2.797 | 8.106 | 2.898 | 2.739 | pseudogamy, endosperm with one unreduced or two reduced cells |
| 5 | 2.891 | 9.417 | 3.258 | | pseudogamy, a sperm cell from triploid plant |
| 5 | 2.781 | 6.749 | 2.427 | | pseudogamy, endosperm with one reduced sperm cell |
| 5 | 2.853 | 6.882 | 2.412 | | pseudogamy, endosperm with one reduced sperm cell |
| 5 | 2.807 | 6.749 | 2.405 | | pseudogamy, endosperm with one reduced sperm cell |
| 6 | 2.773 | 6.784 | 2.446 | 2.773 | pseudogamy, endosperm with one reduced sperm cell |
| 6 | 2.814 | 6.911 | 2.456 | | pseudogamy, endosperm with one reduced sperm cell |
| 6 | 2.834 | 9.786 | 3.453 | | pseudogamy, a sperm cell from triploid plant |
| 6 | 2.852 | 6.966 | 2.443 | | pseudogamy, endosperm with one reduced sperm cell |

| | | | | | |
|---|---|---|---|---|---|
| 6 | 5.568 | 8.295 | 1.49 | | sexual fertilisation of unreduced embryo-sac |
| 6 | 2.869 | 13.469 | 4.694 | | pseudogamy, two sperm cells from triploid plant |
| 7 | 2.822 | 8.257 | 2.926 | 2.901 | pseudogamy, endosperm with one unreduced or two reduced cells |
| 7 | 2.85 | 9.274 | 3.255 | | pseudogamy, a sperm cell from triploid plant |
| 7 | 4.233 | 10.996 | 2.598 | | pseudogamy, triploid embryo, endosperm with one unreduced or two reduced cells |
| 7 | 2.803 | 6.855 | 2.446 | | pseudogamy, endosperm with one reduced sperm cell |
| 7 | 2.799 | 6.886 | 2.46 | | pseudogamy, endosperm with one reduced sperm cell |
| 8 | 2.794 | 9.599 | 3.435 | 2.81 | pseudogamy, a sperm cell from triploid plant |
| 8 | 2.795 | 6.877 | 2.46 | | pseudogamy, endosperm with one reduced sperm cell |
| 8 | 2.784 | 6.847 | 2.46 | | pseudogamy, endosperm with one reduced sperm cell |
| 8 | 2.816 | 6.855 | 2.435 | | pseudogamy, endosperm with one reduced sperm cell |
| 8 | 2.821 | 6.834 | 2.423 | | pseudogamy, endosperm with one reduced sperm cell |
| 9 | 2.823 | 6.952 | 0.406 | 2.801 | pseudogamy, endosperm with one reduced sperm cell |
| 9 | 2.822 | 6.888 | 0.41 | | pseudogamy, endosperm with one reduced sperm cell |
| 9 | 2.797 | 6.919 | 0.404 | | pseudogamy, endosperm with one reduced sperm cell |
| 9 | 2.834 | 6.869 | 0.413 | | pseudogamy, endosperm with one reduced sperm cell |
| 9 | 2.836 | 6.934 | 0.409 | | pseudogamy, endosperm with one reduced sperm cell |
| 9 | 2.818 | 8.329 | 0.338 | | pseudogamy, tetraploid embryo, endosperm with one unreduced or two reduced cells |
| 10 | 2.836 | 6.974 | 0.407 | 2.802 | pseudogamy, endosperm with one reduced sperm cell |
| 10 | 2.822 | 6.973 | 0.405 | | pseudogamy, endosperm with one reduced sperm cell |
| 10 | 2.795 | 6.904 | 0.405 | | pseudogamy, endosperm with one reduced sperm cell |
| 10 | 2.855 | 5.656 | 0.505 | | autonomous apomixis |
| 10 | 2.802 | 8.242 | 0.34 | | pseudogamy, tetraploid embryo, endosperm with one unreduced or two reduced cells |
| 11 | 2.797 | 6.876 | 0.407 | 2.806 | pseudogamy, endosperm with one reduced sperm cell |
| 11 | 2.785 | 8.066 | 0.345 | | pseudogamy, tetraploid embryo, endosperm with one unreduced or two reduced cells |
| 11 | 2.824 | 9.669 | 0.292 | | pseudogamy, a sperm cell from triploid plant |
| 11 | 2.843 | 9.492 | 0.3 | | pseudogamy, a sperm cell from triploid plant |
| 11 | 2.835 | 9.623 | 0.295 | | pseudogamy, a sperm cell from triploid plant |
| 12 | 2.811 | 6.853 | 0.41 | 2.85 | pseudogamy, endosperm with one reduced sperm cell |
| 12 | 2.811 | 6.839 | 0.411 | | pseudogamy, endosperm with one reduced sperm cell |
| 12 | 2.829 | 6.989 | 0.405 | | pseudogamy, endosperm with one reduced sperm cell |
| 12 | 2.783 | 8.168 | 0.341 | | pseudogamy, tetraploid embryo, endosperm with one unreduced or two reduced cells |
| 12 | 2.783 | 6.853 | 0.406 | | pseudogamy, endosperm with one reduced sperm cell |
| 13 | 2.827 | 6.936 | 0.408 | 2.827 | pseudogamy, endosperm with one reduced sperm cell |
| 13 | 2.823 | 6.954 | 0.406 | | pseudogamy, endosperm with one reduced sperm cell |
| 13 | 2.825 | 6.839 | 0.413 | | pseudogamy, endosperm with one reduced sperm cell |
| 13 | 2.819 | 6.958 | 0.405 | | pseudogamy, endosperm with one reduced sperm cell |
| 13 | 2.819 | 11.01 | 0.256 | | pseudogamy, endosperm with two unreduced sperm cells |

**Supplementary Table S3**: Summarized description of localities containing date of collection, elevation, and GPS coordinates.

| locality | date | elevation | GPS N | GPS E | species |
|---|---|---|---|---|---|
| 3 | 25.8.2009 | 5612 | 32.99933 | 78.43314 | *Thylacospermum caespitosum* |
| 3 | 14.8.2013 | 5308 | 33.50514 | 77.76550 | *Saussurea gnaphalodes* |
| 4 | 25.8.2009 | 5664 | 32.99878 | 78.43294 | *Arenaria bryophylla*<br>*Astragalus confertus*<br>*Eritrichum hemisphaericum*<br>*Hedinia tibetica*<br>*Kobresia schoenoides*<br>*Nepeta longibracteata*<br>*Oxytropis chiliophylla*<br>*Oxytropis tatarica*<br>*Poa attenuata*<br>*Potentilla pamirica*<br>*Saussurea gnaphalodes*<br>*Trisetum spicatum*<br>*Waldheimia tridactylites* |
| 4 | 14.8.2013 | 4692 | 33.21561 | 77.55172 | *Chamaerhodos sabulosa*<br>*Senecio dubitabilis* |
| 5 | 25.8.2009 | 5333 | 32.99556 | 78.41606 | *Saussurea hypsipeta* |
| 6 | 25.8.2009 | 5975 | 32.99733 | 78.46233 | *Desideria pumila*<br>*Draba oreades*<br>*Saxifraga nanella* |
| 6 | 14.8.2013 | 5100 | 33.10270 | 77.63190 | *Desideria himalayensis* |
| 7 | 25.8.2009 | 4600 | 32.98558 | 78.37489 | *Hedinia tibetica*<br>*Waldheimia tridactylites* |
| 8 | 25.8.2009 | 5386 | 32.93817 | 78.22036 | *Cremanthodium ellisii*<br>*Urtica hyperborea* |
| 8 | 15.8.2013 | 4309 | 32.91022 | 77.58368 | *Semenovia millefolia* |
| 9 | 25.8.2009 | 5339 | 32.99242 | 78.41039 | *Delphinium brunonianum*<br>*Oxytropis microphylla*<br>*Stipa subsessiliflora* |
| 9 | 15.8.2013 | 4337 | 32.92297 | 77.55135 | *Corydalis stricta*<br>*Eritrichum fruticulosum*<br>*Thymus linearis* |
| 10 | 15.8.2013 | 4341 | 32.92348 | 77.54813 | *Aquilegia moorcroftiana*<br>*Lindelofia stylosa* |
| 11 | 25.8.2009 | 4771 | 32.92667 | 78.25703 | *Physochlaina praealta* |
| 12 | 25.8.2009 | 5436 | 32.99608 | 78.42014 | *Urtica hyperborea*<br>*Astragalus nivalis*<br>*Gentianella moorcroftiana*<br>*Psychrogeton andryaloides* |

| | | | | | |
|---|---|---|---|---|---|
| | | | | | *Psychrogeton denudatus* |
| 16 | 18.8.2013 | 4488 | 32.97847 | 77.42243 | *Alopecurus himalaicus*<br>*Carex stenocarpa* |
| 18 | 18.8.2013 | 4751 | 33.00424 | 77.35732 | *Allium stoliczkae*<br>*Draba cachemirica* |
| 20 | 19.8.2013 | 4995 | 33.02831 | 77.34503 | *Cremanthodium elisii*<br>*Epilobium latifolium* |
| 25 | 20.8.2013 | 4752 | 33.10077 | 77.28547 | *Biebersteinia odora*<br>*Bistorta affinis*<br>*Geranium himalayense*<br>*Potentilla evestita* |
| 26 | 20.8.2013 | 4716 | 33.10103 | 77.27885 | *Nepeta glutinosa* |
| 27 | 20.8.2013 | 4668 | 33.10222 | 77.27527 | *Astragalus oplites*<br>*Caragana sp.*<br>*Elymus schrenkianus*<br>*Silene moorcroftiiana* |
| 31 | 20.8.2013 | 4327 | 33.12469 | 77.23027 | *Heracleum pinnatum* |
| 32 | 20.8.2013 | 3971 | 33.12719 | 77.21786 | *Dracocephalum stamineum* |
| 33 | 21.8.2013 | 3920 | 33.17703 | 77.17185 | *Epilobium latifolium*<br>*Pedicularis bicornuta* |
| 34 | 22.8.2013 | 3831 | 33.23498 | 77.14468 | *Platnago depresa* |
| 39 | 24.8.2013 | 4049 | 33.97122 | 76.33810 | *Potentilla gelida* |
| 42 | 24.8.2013 | 3730 | 34.08401 | 76.04742 | *Lappula tadshikorum*<br>*Scrophularia dentata* |
| 43 | 24.8.2013 | 3469 | 34.06869 | 75.93287 | *Astragalus falconeri* |
| 44 | 24.8.2013 | 3364 | 34.08128 | 75.93463 | *Iris hookeriana* |
| 45 | 25.8.2013 | 3120 | 34.42425 | 76.28330 | *Allium przewalskianum* |
| 46 | 25.8.2013 | 3848 | 34.38103 | 76.46117 | *Corydalis flabellata*<br>*Cousinia thomsonii*<br>*Morina coulteriana* |
| 48 | 25.8.2013 | 3026 | 34.31020 | 76.95679 | *Cicer arietinum*<br>*Matthiola chorassanica* |
| 49 | 27.8.2013 | 5409 | 34.27762 | 77.60632 | *Primula macrophylla* |
| 50 | 27.8.2013 | 5315 | 34.28995 | 77.59465 | *Silene gonosperma* |
| 51 | 27.8.2013 | 5001 | 34.30964 | 77.61867 | *Artemisia minor*<br>*Saussurea leontodontoides* |
| 58 | 28.8.2013 | 3005 | 34.77983 | 77.11216 | *Colutea nepalensis* |
| 60 | 28.8.2013 | 3072 | 34.72409 | 77.20242 | *Capparis spinosa*<br>*Lepidium latifolium* |
| 62 | 28.8.2013 | 4818 | 34.25406 | 77.62244 | *Pedicularis cheilanthifolia* |
| 64 | 2.9.2013 | 5147 | 32.98943 | 78.39602 | *Dracocephallum heterophyllum* |
| 65 | 3.9.2013 | 5410 | 33.10611 | 78.38923 | *Astragalus munroi* |
| 69 | 5.9.2013 | 5741 | 33.05447 | 78.43301 | *Pegaeophyton scapiflorum* |
| 70 | 7.9.2013 | 5636 | 32.99562 | 78.42884 | *Arenaria bryophylla*<br>*Delphinium brunonianum*<br>*Nepeta longibracteata* |

| | | | | | |
|---|---|---|---|---|---|
| | | | | | *Oxytropis tatarica* |
| | | | | | *Potentilla pamirica* |
| | | | | | *Saussurea glacialis* |
| | | | | | *Saussurea gnaphaloides* |
| | | | | | *Saxifraga hirculus* |
| | | | | | *Trisetum spicatum* |
| | | | | | *Waldheimia tridactylites* |
| 71 | 7.9.2013 | 5355 | 32.99213 | 78.41049 | *Saussurea medusa* |
| 72 | 7.9.2013 | 5302 | 32.99302 | 78.41013 | *Oxytropis chiliophylla* |
| | | | | | *Saussurea glandulifera* |
| 73 | 7.9.2013 | 5423 | 32.99544 | 78.41605 | *Cremanthodium elisii* |
| 74 | 7.9.2013 | 5147 | 32.98943 | 78.39602 | *Artemisia santolinifolia* |
| | | | | | *Artemisia stracheyi* |
| | | | | | *Oxytropis microphylla* |
| | | | | | *Stipa subsessiliflora* |
| 75 | 8.9.2013 | 4698 | 32.98358 | 78.35858 | *Artemisia demissa* |
| | | | | | *Artemisia moorcroftiana* |
| | | | | | *Braya humilis* |
| | | | | | *Corispermum tibeticum* |
| | | | | | *Crepis flexuosa* |
| | | | | | *Christolea crassifolia* |
| | | | | | *Knorringia pamirica* |
| | | | | | *Kobresia schoenoides* |
| | | | | | *Krascheninnikovia pungens* |
| | | | | | *Physochlaina praealta* |
| | | | | | *Silene nepalensis* |
| | | | | | *Stevenia canescens* |
| | | | | | *Tanacetum fruticulosum* |
| 204 | 8.9.2013 | 4698 | 32.98358 | 78.35858 | *Potentilla bifutca* |
| 290 | 4.9.2013 | NA | NA | NA | *Potentilla atrosanguinea* |
| 764 | 5.9.2013 | 5780 | 33.01263 | 78.44185 | *Desideria pumila* |
| 765 | 6.9.2013 | 5650 | 33.00042 | 78.43462 | *Ranunculus lobatus* |
| 05-9-44 | 13.8.2005 | 3500 | NA | NA | *Elymus schugnanicus* |
| 06-1-7 | 5.8.2006 | 3480 | 34.16017 | 77.58117 | *Eremopoa altaica* |
| 04-11-9 | 16.8.2004 | 4650 | 36.93417 | 77.23350 | *Carex stenocarpa* |
| 06-8-10 | 12.8.2006 | 5000 | 34.41583 | 77.31083 | *Poa attenuata* |
| 06-8-13 | 12.8.2006 | 4850 | 34.43650 | 77.32250 | *Puccinellia ladakhensis* |
| 05-2-16 | 6.8.2005 | 3100 | 34.42667 | 75.75667 | *Bromus oxyodon* |
| 05-11-17 | 18.5.2005 | 3220 | 34.37933 | 75.59333 | *Phalaris canariensis* |
| 01-34-15 | 1.9.2001 | 4520 | 33.37833 | 77.75167 | *Stipa koelzii* |
| 01-38-45 | 4.9.2001 | 4370 | 23.60667 | 77.76333 | *Stipa mongolica* |
| 01-45-13 | 19.9.2001 | 4500 | 34.06333 | 77.82667 | *Poa pratensis ssp. staintonii* |
| 02-31-9 | 13.8.2002 | 2850 | 34.51167 | 76.62667 | *Enneapogon persicus* |
| 02-45-13 | 15.9.2002 | 4050 | 33.99833 | 77.83333 | *Catabrosa aquatica* |
| 03-20-11 | 31.8.2003 | 4770 | 34.68167 | 76.71500 | *Poa alpina* |
| 03-34-1 | 16.9.2003 | NA | NA | NA | *Stellaria depressa* |

| | | | | | |
|---|---|---|---|---|---|
| 03-39-4 | 21.9.2003 | 5100 | 33.69833 | 78.35333 | *Festuca nitidula* |
| 03-41-9 | 23.9.2003 | 4630 | 34.81167 | 78.35500 | *Trikeraia oreophila* |
| 04-20-12 | 25.8.2004 | 4200 | 33.40750 | 77.23750 | *Bromus tectorum* |
| 04-27-23 | 1.9.2004 | 4300 | 33.84967 | 76.37350 | *Elymus dentatus* |
| 04-29-13a | 3.9.2004 | 4100 | 33.98167 | 76.35500 | *Festuca wallichiana* |
| 04-36-12 | 18.4.2004 | 3000 | 34.27817 | 75.98100 | *Digitaria stewartiana* |
| 04-38-39 | 12.9.2004 | 3400 | 34.40100 | 75.74667 | *Poa suruana* |
| 04-40-14 | 14.9.2004 | 3200 | 34.42667 | 75.75667 | *Poa sterilis* |
| 04-40-16 | 14.9.2004 | 3370 | 34.42400 | 75.76383 | *Poa pratensis ssp. pratensis* |
| 04-40-19 | 14.9.2004 | 3600 | 34.41350 | 75.79217 | *Phleum alpinum* |
| 04-44-64 | 18.9.2004 | 2680 | 34.56500 | 76.13333 | *Echinochloa crus-galli* |
| 04-48-2 | 23.9.2004 | 5100 | NA | NA | *Carex borii* |
| 04-55-9 | 30.9.2003 | 3700 | NA | NA | *Stipa splendens* |
| 05-30-13a | 3.9.2005 | 5100 | 49.50050 | 78.30583 | *Festuca non-coelestis* |
| 05-30-13b | 3.9.2005 | 5200 | NA | NA | *Festuca coelestis* |
| 05-34-25 | 7.9.2005 | 4610 | 33.33100 | 78.03650 | *Puccinellia stapfiana* |
| 05-35-3 | 8.9.2005 | 4540 | 33.29083 | 78.02383 | *Puccinellia pauciramea* |
| 05-44-25 | 14.5.2005 | 4100 | NA | NA | *Carex stenophylla* |
| 06-20-11 | 24.8.2006 | 4600 | 34.48667 | 77.95183 | *Poa attenuata* |
| 99-1-22 | 13.8.1999 | 3650 | 34.16667 | 77.58333 | *Eragrostis minor* |
| H25-4 | 2015 | NA | NA | NA | *Stipa splendens* |
| K224 | 20.8.2014 | 4556 | 33.10202 | 77.76091 | *Elymus jacquemontii* |
| | | | | | *Potentilla bifurca* |
| K225 | 20.8.2014 | 5117 | 33.10362 | 77.63248 | *Thylacospermum caespitosum* |
| K226 | 20.8.2014 | 4942 | 33.06463 | 77.63195 | *Kobresia macrantha* |
| K227 | 20.8.2014 | 4716 | 32.81374 | 77.45328 | *Biebersteinia odora* |
| | | | | | *Polygonum cognatum* |
| K228 | 20.8.2014 | 4927 | 32.75915 | 77.41944 | *Carex nivalis* |
| | | | | | *Eritrichum villosum* |
| K229 | 21.8.2014 | 3938 | 32.80223 | 77.11360 | *Carex nivalis* |
| | | | | | *Chenopodium foliosum* |
| | | | | | *Juncus himalensis* |
| | | | | | *Juncus leucanthus* |
| | | | | | *Myricaria germanica* |
| | | | | | *Oxyria digyna* |
| | | | | | *Sibbaldia cuneata* |
| K230 | 21.8.2014 | 4021 | 32.79939 | 77.11203 | *Leontopodium leontopodinum* |
| | | | | | *Potentilla sojakii* |
| | | | | | *Sagina saginoides* |
| K231 | 22.8.2014 | 3960 | 32.80444 | 77.11590 | *Brachyactis roylei* |
| | | | | | *Erigeron venustus* |
| | | | | | *Galium pauciflorum* |
| | | | | | *Lindelofia anchusoides* |
| | | | | | *Veronica biloba* |
| K232 | 22.8.2014 | 4186 | 32.81303 | 77.12123 | *Carex moorcroftii* |
| | | | | | *Cicer microphyllum* |

| | | | | | |
|---|---|---|---|---|---|
| | | | | | *Phleum alpinum* |
| | | | | | *Rheum webbianum* |
| K234 | 22.8.2014 | 4438 | 32.83256 | 77.15257 | *Anaphalis nubigena* |
| | | | | | *Arabis tibetica* |
| | | | | | *Potentilla venusta* |
| K235 | 23.8.2014 | 4571 | 32.86123 | 77.18407 | *Aster flaccidus* |
| | | | | | *Corydalis thyrsiflora* |
| | | | | | *Draba lasiophylla* |
| | | | | | *Draba sp.* |
| | | | | | *Picrorhiza kurrooa* |
| | | | | | *Pleurospermum lindleyanum* |
| | | | | | *Salix flabellaris* |
| | | | | | *Silene himalayensis* |
| | | | | | *Silene tenuis* |
| K236 | 23.8.2014 | 4617 | 32.86792 | 77.18644 | *Draba cachemirica* |
| | | | | | *Meconopsis aculeata* |
| K239 | 23.8.2014 | 5069 | 32.90885 | 77.20044 | *Aphragmus oxycarpus* |
| | | | | | *Draba sp.* |
| K240 | 23.8.2014 | 4721 | 32.92509 | 77.21188 | *Carex borii* |
| | | | | | *Ranunculus membranaceus* |
| K241 | 23.8.2014 | 4720 | 32.92857 | 77.22148 | *Erigeron uniflorus* |
| | | | | | *Meconopsis aculeata* |
| | | | | | *Potentilla sojakii* |
| | | | | | *Potentilla turczaninowiana* |
| | | | | | *Primula moorcroftiana* |
| K242 | 24.8.2014 | 4720 | 32.93134 | 77.22835 | *Potentilla multifida* |
| | | | | | *Potentilla sericea* |
| | | | | | *Potentilla sojakii* |
| | | | | | *Potentilla venusta* |
| | | | | | *Saussurea schulzii* |
| | | | | | *Silene tenuis* |
| K243 | 24.8.2014 | 4630 | 32.93208 | 77.23458 | *Rhodiola heterodonta* |
| K244 | 24.8.2014 | 4611 | 32.93400 | 77.23645 | *Astragalus rhizanthus* |
| K245 | 24.8.2014 | 4520 | 32.93704 | 77.23843 | *Arabis pauciflora* |
| | | | | | *Polygonum rumicifolium* |
| | | | | | *Sisymbrium brassiciforme* |
| K246 | 24.8.2014 | 4359 | 32.96944 | 77.25015 | *Astragalus tecti-mundi* |
| K247 | 25.8.2014 | 4132 | 33.06148 | 77.22781 | *Astragalus thomsonii* |
| | | | | | *Cuscuta planiflora* |
| | | | | | *Oxytropis hypoglottoides* |
| | | | | | *Rubia tibetica* |
| K248 | 25.8.2014 | 4072 | 33.07146 | 77.21993 | *Rubia tibetica* |
| | | | | | *Scorzonera virgata* |
| | | | | | *Semenovia lasiocarpa* |
| K249 | 25.8.2014 | 4051 | 33.07653 | 77.21663 | *Hordeum brevisubulatum* |
| | | | | | *Leymus secalinus* |

| | | | | | |
|---|---|---|---|---|---|
| | | | | | *Pedicularis cheilanthifolia*<br>*Plantago himalaica*<br>*Scorzonera virgata*<br>*Tragopogon gracilis* |
| K251 | 25.8.2014 | 4053 | 33.10115 | 77.21462 | *Acantholimon lycopodioides*<br>*Lonicera asperifolia* |
| K252 | 25.8.2014 | 4053 | 33.12365 | 77.21821 | *Kochia prostrata*<br>*Stipa orientalis* |
| K254 | 26.8.2014 | 3970 | 33.14684 | 77.20056 | *Cynanchum acutum*<br>*Rhamnus prostrata*<br>*Scrophularia dentata* |
| K255 | 26.8.2014 | 3969 | 33.15542 | 77.19190 | *Calamagrostis stoliczkae* |
| K256 | 26.8.2014 | 4005 | 33.15542 | 77.19190 | *Atriplex pamirica*<br>*Conringia planisiliqua* |
| K257 | 26.8.2014 | 3960 | 33.16600 | 77.18083 | *Arnebia euchroma*<br>*Carex orbicularis*<br>*Conioselinum vaginatum*<br>*Euphrasia foliosa*<br>*Gentianella moorcroftiana*<br>*Juncus memranaceus*<br>*Rumex patientia*<br>*Thalictrum foetidum* |
| K258 | 25.8.2014 | 3995 | 33.17729 | 77.16746 | *Kochia prostrata* |
| K259 | 26.8.2014 | 3962 | 33.18758 | 77.16001 | *Elymus cognatus*<br>*Scrophularia dentata*<br>*Sisymbrium brassiciforme* |
| K260 | 26.8.2014 | 3910 | 33.20061 | 77.15382 | *Heracleum pinnatum* |
| K262 | 27.8.2014 | 3832 | 33.24678 | 77.16468 | *Clemaatis tangutica*<br>*Ribes orientale* |
| K263 | 27.8.2014 | 3822 | 33.25159 | 77.16686 | *Lonicera microphylla* |
| K264 | 27.8.2014 | 3824 | 33.25917 | 77.17309 | *Anthemis sp.* |
| K265 | 27.8.2014 | 3867 | 33.26434 | 77.17808 | *Euphorbia tibetica*<br>*Melica persica*<br>*Scrophularia dentata* |
| K266 | 27.8.2014 | 3839 | 33.23953 | 77.10462 | *Stipa splendens* |
| K268 | 30.8.2014 | 3414 | 33.71141 | 76.88908 | *Saussurea jacea*<br>*Sorbus tianschanica* |
| K269 | 30.8.2014 | 3415 | 33.71969 | 76.86824 | *Leptorhabdos parviflora*<br>*Scrophularia dentata* |
| K270 | 31.8.2014 | 3417 | 33.72277 | 76.85841 | *Scrophularia dentata* |
| K271 | 31.8.2014 | 3459 | 33.72719 | 76.86253 | *Bupleurum gracillimum*<br>*Scrophularia dentata* |
| K273 | 31.8.2014 | 3394 | 33.73200 | 76.85768 | *Clematis sp.*<br>*Comarum salesovianum* |
| K274 | 31.8.2014 | 3688 | 33.77235 | 76.84057 | *Rosa webbiana* |
| K279 | 1.9.2014 | 3761 | 33.80561 | 76.82697 | *Scrophularia dentata* |

| | | | | | |
|---|---|---|---|---|---|
| K280 | 1.9.2014 | 3989 | 33.81665 | 76.81720 | *Anemone rupicola*<br>*Aquilegia fragrans* |
| K281 | 1.9.2014 | 3991 | 33.81822 | 76.81636 | *Aquilegia moorcroftiana*<br>Brassicaceae |
| K282 | 1.9.2014 | 4033 | 33.81888 | 76.81567 | *Askellia naniformis* |
| K283 | 1.9.2014 | 4218 | 33.82450 | 76.81200 | *Rheum tibeticum* |
| K284 | 1.9.2014 | 4415 | 33.84740 | 76.79496 | *Thermopsis inflata* |
| K285 | 1.9.2014 | 4762 | 33.87130 | 76.79153 | *Biebersteinia odora*<br>*Parrya nudicaulis*<br>*Valeriana himalayana* |
| K286 | 1.9.2014 | 4110 | 33.88391 | 76.79256 | *Marmoritis rotundifolia* |
| K287 | 1.9.2014 | 4235 | 33.89228 | 76.79030 | *Cerastium sp.* |
| K288 | 2.9.2014 | 3964 | 33.90622 | 76.83012 | *Clematis sp.*<br>*Tauscheria lasiocarpa* |
| K301 | 6.7.1905 | NA | NA | NA | *Batrachium flavescens* |
| K3019 | 6.7.1905 | NA | NA | NA | *Hedinia tibetica* |
| K311 | 9.9.2014 | 4998 | 33.13796 | 78.37360 | *Lepidium capitatum* |
| K313 | 10.9.2014 | 5265 | 33.11692 | 78.38064 | *Carex microglochin*<br>*Halerpestes lancifolia* |
| K316 | 10.9.2014 | 5412 | 33.10357 | 78.38951 | *Saussurea andryaloides* |
| K317 | 10.9.2014 | 5440 | 33.09971 | 78.39152 | *Saussurea bracteata* |
| K318 | 11.9.2014 | 5539 | 33.09537 | 78.39410 | *Draba sp.* |
| K318 | 11.9.2014 | 5539 | 33.09537 | 78.39410 | *Trisetum spicatum* |
| K319 | 11.9.2014 | 5603 | 33.07464 | 78.40282 | *Potentilla pamirica* |
| K320 | 2014 | NA | NA | NA | *Stellaria depressa* |
| K331 | 13.9.2014 | 5631 | 32.99877 | 78.42861 | *Poa attenuata* |
| K333 | 13.9.2014 | 5356 | 32.99330 | 78.41172 | *Rhodiola heterodonta*<br>*Eritrichum hemisphaericum* |
| K339 | 13.9.2014 | 5356 | 32.99330 | 78.41172 | *Saxifraga hirculoides* |
| K341 | 13.9.2014 | 5153 | 32.98983 | 78.39714 | *Alyssum canescens* |
| K363 | 14.9.2014 | 4571 | 33.03300 | 78.27468 | *Corispermum tibeticum*<br>*Chenopodium pamiricum*<br>*Salsola jacquemontii*<br>*Senecio dubitabilis* |
| K435 | NA | NA | NA | NA | *Saussurea medusa* |
| Lagong | 24.8.2015 | 4400 | NA | NA | *Festuca olgae* |
| MK 2 | 27.8.2013 | 4475 | 34.18061 | 76.66306 | *Pulsatilla wallichiana* |
| MK 5 | 27.8.2013 | 4209 | 34.16908 | 76.69171 | *Comarum salesovianum* |
| MK 9 | 27.8.2013 | 3666 | 34.20951 | 76.72616 | *Tanacetum stoliczkae* |
| TR1 | 27.8.2013 | 3735 | 33.90987 | 77.70122 | *Gentianopsis vvedenskyi*<br>*Nepeta leucolaena* |
| TR13 | 11.9.2013 | 2268 | 32.26203 | 77.17497 | *Geranium nepalense* |
| TR15 | 13.9.2013 | 2721 | 32.20002 | 77.24602 | *Impatiens brachycentra* |
| TR22 | 16.9.2013 | 2211 | 32.10797 | 77.19031 | *Lecanthus peduncularis* |
| TR3 | 31.8.2013 | 3633 | 32.68439 | 77.18882 | *Dianthus harrissii*<br>*Nepeta eriostachya* |

| | | | | | |
|---|---|---|---|---|---|
| TR4 | 31.8.2013 | 3365 | 32.67487 | 77.19789 | *Himalaiella albescens* |
| | | | | | *Medicago falcata* |
| | 2011 | NA | NA | NA | *Ladakiella klimesii* |
| | 14.8.2013 | 3810 | 33.44113 | 77.44338 | *Stachys tibetica* |
| | 15.8.2013 | 4329 | 32.92427 | 77.54567 | *Nepeta discolor* |
| | 17.8.2013 | 4407 | 32.94226 | 77.48449 | *Cicer microphyllum* |
| | 17.8.2013 | 4407 | 32.94226 | 77.48449 | *Stipa capilata* |
| | 18.8.2013 | 4488 | 32.97847 | 77.42243 | *Oxytropis pussila* |
| | 21.8.2013 | 3920 | 33.17703 | 77.17185 | *Artemisia hedinii* |

**Supplementary Table S4**: Summary of analysed species with information about family, number of measured seeds, used FCSS protocol, information about way of reproduction, avarage embryo / endosperm ratio, percentage of endospermatic nuclei in a seed, and altitudinal optimim. Reproduction systems are symbolized by colours: yellow colour indicates sexual reproduction (sexual), green signals apomictic reproduction (apomictic), and grey belongs to species with undetectable endosperm (×).

| family | species | no. of measured seeds | FCSS protocol | reproduction systém | embryo / endosperm ratio | percentage of endosperm | altitudinal optimum |
|---|---|---|---|---|---|---|---|
| Alliaceae | *Allium przewalskianum* | 20 | M | sexual | 1.46 | 50.98 | 4069 |
| Amaranthaceae | *Salsola jacquemontii* | 9 | M | sexual | 1.48 | 1.47 | 4524 |
| Apiaceae | *Bupleurum gracillimum* | 9 | M | sexual | 1.57 | 80.14 | 3932 |
| Apiaceae | *Conioselinum vaginatum* | 13 | M | sexual | 1.55 | 57.40 | 3669 |
| Apiaceae | *Heracleum pinnatum* | 26 | M | sexual | 1.52 | 76.06 | 3971 |
| Apiaceae | *Pleurospermum lindleyanum* | 6 | O | sexual | 1.60 | 81.05 | 4948 |
| Apiaceae | *Semenovia lasiocarpa* | 8 | O | sexual | 1.57 | 91.04 | 3994 |
| Apiaceae | *Semenovia millefolia* | 3 | O | × | × | × | 3994 |
| Apocynaceae | *Cynanchum acutum* | 13 | O | sexual | 1.48 | 41.63 | 2939 |
| Asteraceae | *Anaphalis nubigena* | 15 | O | sexual | 1.46 | 35.09 | 4017 |
| Asteraceae | *Anthemis cotula* | 10 | O | sexual | 1.48 | 11.27 | 3415 |
| Asteraceae | *Artemisia demissa* | 9 | O | sexual | 1.51 | 12.10 | 4355 |
| Asteraceae | *Artemisia hedinii* | 10 | O | sexual | 1.50 | 11.21 | 4426 |
| Asteraceae | *Artemisia minor* | 10 | M | sexual | 1.52 | 4.74 | 5069 |
| Asteraceae | *Artemisia moorcroftiana* | 10 | O | sexual | 1.49 | 13.91 | 3269 |
| Asteraceae | *Artemisia santolinifolia* | 13 | O | sexual | 1.47 | 25.06 | 4784 |
| Asteraceae | *Artemisia stracheyi* | 9 | O | sexual | 1.48 | 14.85 | 4962 |
| Asteraceae | *Askellia naniformis* | 6 | O | sexual | 1.45 | 13.74 | 4800 |
| Asteraceae | *Aster flaccidus* | 13 | O | sexual | 1.46 | 18.11 | 5271 |
| Asteraceae | *Brachyactis roylei* | 10 | O | sexual | 1.48 | 24.44 | 3726 |
| Asteraceae | *Cousinia thomsonii* | 10 | O | sexual | 1.45 | 11.43 | 3523 |
| Asteraceae | *Cremanthodium ellisii* | 19 | O | sexual | 1.47 | 27.93 | 5061 |
| Asteraceae | *Crepis flexuosa* | 9 | M | sexual | 1.50 | 8.76 | 4447 |
| Asteraceae | *Erigeron uniflorus* | 15 | O | sexual | 1.45 | 12.22 | 4209 |
| Asteraceae | *Erigeron venustus* | 20 | O | sexual | 1.48 | 28.99 | NA |
| Asteraceae | *Himalaiella albescens* | 10 | O | sexual | 1.48 | 14.02 | 3360 |
| Asteraceae | *Leontopodium leontopodinum* | 15 | O | sexual | 1.45 | 20.51 | 4696 |
| Asteraceae | *Psychrogeton andryaloides* | 10 | O | sexual | 1.49 | 8.88 | 4264 |
| Asteraceae | *Psychrogeton denudatus* | 10 | O | sexual | 1.49 | 13.00 | 4233 |
| Asteraceae | *Saussurea andryaloides* | 3 | M | sexual | 1.46 | 19.61 | 4905 |
| Asteraceae | *Saussurea bracteata* | 6 | M | × | × | × | 5271 |
| Asteraceae | *Saussurea glacialis* | 11 | O | sexual | 1.47 | 16.08 | 5542 |
| Asteraceae | *Saussurea glanduligera* | 6 | O | sexual | 1.43 | 16.98 | 5082 |

| | | | | | | | |
|---|---|---|---|---|---|---|---|
| Asteraceae | *Saussurea gnaphalodes* | 19 | O | sexual | 1.47 | 14.35 | 5843 |
| Asteraceae | *Saussurea hypsipeta* | 14 | M | sexual | 1.40 | 6.95 | 5853 |
| Asteraceae | *Saussurea jacea* | 3 | O | × | × | × | 3856 |
| Asteraceae | *Saussurea leontodontoides* | 10 | O | sexual | 1.48 | 12.39 | 4813 |
| Asteraceae | *Saussurea medusa* | 10 | O | × | × | × | 5548 |
| Asteraceae | *Saussurea schultzii* | 13 | O | × | × | × | 4713 |
| Asteraceae | *Scorzonera virgata* | 12 | O | sexual | 1.49 | 10.32 | 3996 |
| Asteraceae | *Senecio dubitabilis* | 16 | M/O | sexual | 1.47 | 18.64 | 4235 |
| Asteraceae | *Tanacetum fruticulosum* | 10 | O | sexual | 1.51 | 8.50 | 4234 |
| Asteraceae | *Tanacetum stoliczkae* | 6 | O | sexual | 1.41 | 12.57 | 3867 |
| Asteraceae | *Tragopogon gracilis* | 10 | O | sexual | 1.45 | 5.90 | 3504 |
| Asteraceae | *Waldheimia tridactylites* | 21 | M | sexual | 1.45 | 7.90 | 5853 |
| Balsaminaceae | *Impatiens brachycentra* | 13 | M | sexual | 1.45 | 7.89 | 2874 |
| Biebersteiniaceae | *Biebersteinia odora* | 72 | O | apom./sex | 1.49, 2.04, 2.81 | 23.75 | 4789 |
| Boraginaceae | *Arnebia euchroma* | 6 | O | × | × | × | 4300 |
| Boraginaceae | *Eritrichium fruticulosum* | 6 | M | sexual | 1.52 | 9.39 | 4324 |
| Boraginaceae | *Eritrichium hemisphaericum* | 8 | M | sexual | 1.57 | 9.67 | 5636 |
| Boraginaceae | *Eritrichium villosum* | 10 | M | × | × | × | 4583 |
| Boraginaceae | *Lappula tadshikorum* | 10 | O | sexual | 1.52 | 16.08 | 3773 |
| Boraginaceae | *Lindelofia anchusoides* | 10 | O | sexual | 1.55 | 5.54 | 3554 |
| Boraginaceae | *Lindelofia stylosa* | 10 | O | × | × | × | 4376 |
| Brassicaceae | *Alyssum canescens* | 12 | M | sexual | 1.43 | 17.47 | 4767 |
| Brassicaceae | *Aphragmus oxycarpus* | 9 | M | sexual | 1.46 | 17.12 | 5481 |
| Brassicaceae | *Arabis paniculata* | 5 | O | × | × | × | 3630 |
| Brassicaceae | *Arabis pauciflora* | 6 | M | sexual | 1.49 | 12.24 | NA |
| Brassicaceae | *Arabis tibetica* | 19 | M | sexual | 1.46 | 14.50 | 4277 |
| Brassicaceae | Brassicaceae | 3 | O | sexual | 1.48 | 57.55 | NA |
| Brassicaceae | *Braya humilis* | 9 | O | sexual | 1.49 | 18.97 | 4857 |
| Brassicaceae | *Conringia planisiliqua* | 15 | M | × | × | × | 3712 |
| Brassicaceae | *Desideria himalayensis* | 3 | O | sexual | 1.44 | 8.88 | 5498 |
| Brassicaceae | *Desideria pumila* | 19 | M/O | sexual | 1.48 | 18.96 | 5813 |
| Brassicaceae | *Draba cachemirica* | 12 | M | sexual | 1.47 | 10.79 | 4652 |
| Brassicaceae | *Draba lasiophylla* | 22 | M | sexual | 1.43 | 25.66 | 5028 |
| Brassicaceae | *Draba oreades* | 15 | M | sexual | 1.44 | 7.80 | 5808 |
| Brassicaceae | *Draba sp.* | 23 | O | sexual | 1.45 | 8.79 | NA |
| Brassicaceae | *Draba sp.* | 10 | O | sexual | 1.45 | 13.97 | NA |
| Brassicaceae | *Draba sp.* | 5 | O | sexual | 1.47 | 6.29 | NA |
| Brassicaceae | *Hedinia tibetica* | 39 | M | sexual | 1.50 | 6.67 | 5434 |
| Brassicaceae | *Christolea crassifolia* | 10 | O | sexual | 1.48 | 21.77 | 4377 |
| Brassicaceae | *Ladakiella klimesii* | 4 | M | sexual | 1.45 | 6.68 | 5862 |
| Brassicaceae | *Lepidium capitatum* | 9 | M | × | × | × | 4593 |
| Brassicaceae | *Lepidium latifolium* | 13 | M | × | × | × | 3133 |
| Brassicaceae | *Matthiola chorassanica* | 9 | O | sexual | 1.49 | 15.21 | 3248 |
| Brassicaceae | *Parrya nudicaulis* | 9 | M | sexual | 1.47 | 3.94 | 4807 |
| Brassicaceae | *Pegaeophyton scapiflorum* | 13 | M | sexual | 1.44 | 2.21 | 5567 |
| Brassicaceae | *Sisymbrium brassiciforme* | 23 | O | sexual | 1.50 | 2.60 | 3923 |

| | | | | | | | |
|---|---|---|---|---|---|---|---|
| Brassicaceae | *Tauscheria lasiocarpa* | 9 | M | sexual | 1.47 | 1.57 | 3670 |
| Capparaceae | *Capparis spinosa* | 13 | O | sexual | 1.50 | 21.43 | 2832 |
| Caprifoliaceae | *Lonicera asperifolia* | 10 | O | sexual | 1.50 | 86.34 | 4041 |
| Caprifoliaceae | *Lonicera microphylla* | 10 | O | sexual | 1.48 | 74.61 | 4249 |
| Caryophyllaceae | *Arenaria bryophylla* | 29 | M | sexual | 1.42 | 6.01 | 5397 |
| Caryophyllaceae | *Cerastium sp.* | 6 | O | sexual | 1.50 | 8.56 | NA |
| Caryophyllaceae | *Dianthus harrissii* | 9 | O | sexual | 1.44 | 5.61 | 4034 |
| Caryophyllaceae | *Sagina saginoides* | 40 | M | sexual | 1.48 | 10.39 | 3679 |
| Caryophyllaceae | *Silene gonosperma* | 10 | O | sexual | 1.43 | 5.25 | 5441 |
| Caryophyllaceae | *Silene himalayensis* | 12 | M | sexual | 1.47 | 6.72 | 5241 |
| Caryophyllaceae | *Silene moorcroftiana* | 3 | O | sexual | 1.6 | 5.89 | 4041 |
| Caryophyllaceae | *Silene nepalensis* | 10 | O | sexual | 1.45 | 17.85 | 3434 |
| Caryophyllaceae | *Silene tenuis* | 19 | O | sexual | 1.44 | 10.80 | 4038 |
| Caryophyllaceae | *Stellaria depressa* | 6 | M | × | × | × | 5381 |
| Caryophyllaceae | *Thylacospermum caespitosum* | 24 | M/O | sexual | 1.49 | 16.80 | 5461 |
| Crassulaceae | *Rhodiola heterodonta* | 18 | M | × | × | × | 4071 |
| Cuscutaceae | *Cuscuta planiflora* | 9 | M | sexual | 1.54 | 24.06 | 3664 |
| Cyperaceae | *Carex borii* | 15 | M | sexual | 1.58 | 50.12 | 5268 |
| Cyperaceae | *Carex microglochin* | 3 | M | × | × | × | 4721 |
| Cyperaceae | *Carex moorcroftii* | 9 | M | sexual | 1.50 | 58.77 | 4944 |
| Cyperaceae | *Carex nivalis* | 17 | M | sexual | 1.66 | 49.20 | 5075 |
| Cyperaceae | *Carex orbicularis* | 9 | M | sexual | 1.52 | 58.20 | 4459 |
| Cyperaceae | *Carex stenocarpa* | 17 | M | sexual | 1.63 | 33.78 | 4465 |
| Cyperaceae | *Carex stenophylla* | 20 | M | sexual | 1.50 | 45.17 | 4127 |
| Cyperaceae | *Kobresia macrantha* | 12 | O | × | × | × | 4435 |
| Cyperaceae | *Kobresia schoenoides* | 13 | M | sexual | 1.51 | 48.63 | 5235 |
| Euphorbiaceae | *Euphorbia tibetica* | 13 | O | sexual | 1.47 | 60.21 | 4735 |
| Fabaceae | *Astragalus confertus* | 9 | M | sexual | 1.43 | 10.85 | 5422 |
| Fabaceae | *Astragalus falconeri* | 10 | O | sexual | 1.47 | 8.93 | 3514 |
| Fabaceae | *Astragalus munroi* | 10 | O | sexual | 1.46 | 5.05 | 3739 |
| Fabaceae | *Astragalus nivalis* | 10 | O | sexual | 1.47 | 9.71 | 4583 |
| Fabaceae | *Astragalus oplites* | 7 | O | sexual | 1.47 | 8.80 | 4110 |
| Fabaceae | *Astragalus rhizanthus* | 13 | O | sexual | 1.50 | 7.63 | 4254 |
| Fabaceae | *Astragalus tecti-mundi* | 6 | O | × | × | × | 4237 |
| Fabaceae | *Astragalus thomsonii* | 10 | O | sexual | 1.50 | 7.94 | 4122 |
| Fabaceae | *Caragana versicolor* | 9 | O | sexual | 1.45 | 4.10 | 4772 |
| Fabaceae | *Cicer arietinum* | 10 | O | sexual | 1.5 | 3.02 | 3918 |
| Fabaceae | *Cicer microphyllum* | 21 | O | × | × | × | 4240 |
| Fabaceae | *Colutea nepalensis* | 12 | O | sexual | 1.48 | 7.10 | 3258 |
| Fabaceae | *Medicago falcata* | 9 | O | sexual | 1.47 | 8.86 | 3058 |
| Fabaceae | *Oxytropis hypoglottoides* | 10 | O | sexual | 1.45 | 9.18 | 4522 |
| Fabaceae | *Oxytropis chiliophylla* | 18 | M/O | sexual | 1.47 | 7.26 | 5266 |
| Fabaceae | *Oxytropis microphylla* | 34 | O | sexual | 1.47 | 5.97 | 4795 |
| Fabaceae | *Oxytropis pusilla* | 9 | O | sexual | 1.48 | 10.50 | 4663 |
| Fabaceae | *Oxytropis tatarica* | 19 | O | sexual | 1.47 | 6.96 | 5019 |
| Fabaceae | *Thermopsis inflata* | 11 | O | sexual | 1.48 | 3.33 | 4583 |

| | | | | | | | |
|---|---|---|---|---|---|---|---|
| Fumariaceae | *Corydalis flabellata* | 13 | M | sexual | 1.42 | 84.18 | 3509 |
| Fumariaceae | *Corydalis stricta* | 3 | O | sexual | 1.55 | 72.04 | 4640 |
| Fumariaceae | *Corydalis thyrsiflora* | 10 | M | sexual | 1.50 | 37.14 | 4524 |
| Gentianaceae | *Gentianella moorcroftiana* | 15 | O | sexual | 1.48 | 78.32 | 3850 |
| Gentianaceae | *Gentianopsis vvedenskyi* | 10 | O | sexual | 1.51 | 83.23 | 3700 |
| Geraniaceae | *Geranium himalayense* | 13 | M | × | × | × | 4164 |
| Geraniaceae | *Geranium nepalense* | 10 | O | × | × | × | 3085 |
| Grossulariaceae | *Ribes orientale* | 6 | O | sexual | 1.51 | 85.27 | 3251 |
| Chenopodiaceae | *Atriplex pamirica* | 13 | O | × | × | × | 4587 |
| Chenopodiaceae | *Corispermum tibeticum* | 13 | M | sexual | 1.42 | 2.68 | 4197 |
| Chenopodiaceae | *Chenopodium foliosum* | 15 | M | sexual | 1.51 | 2.11 | 3233 |
| Chenopodiaceae | *Chenopodium pamiricum* | 8 | M | sexual | 1.49 | 3.35 | 4644 |
| Chenopodiaceae | *Kochia prostrata* | 13 | O | sexual | 1.47 | 4.06 | 3601 |
| Chenopodiaceae | *Krascheninnikovia pungens* | 6 | O | × | × | × | 4762 |
| Iridaceae | *Iris hookeriana* | 10 | M | sexual | 1.51 | 82.96 | 3480 |
| Juncaceae | *Juncus himalensis* | 25 | M | sexual | 1.50 | 34.35 | 3701 |
| Juncaceae | *Juncus leucanthus* | 25 | M | sexual | 1.47 | 38.65 | 3900 |
| Juncaceae | *Juncus membranaceus* | 15 | M | sexual | 1.52 | 28.77 | 4040 |
| Lamiaceae | *Dracocephalum heterophyllum* | 10 | O | sexual | 1.47 | 9.58 | 4867 |
| Lamiaceae | *Dracocephalum stamineum* | 9 | O | sexual | 1.47 | 16.61 | 4367 |
| Lamiaceae | *Marmoritis rotundifolia* | 10 | O | sexual | 1.50 | 9.89 | 4761 |
| Lamiaceae | *Nepeta discolor* | 10 | O | sexual | 1.49 | 11.15 | 4421 |
| Lamiaceae | *Nepeta eriostachya* | 10 | O | sexual | 1.46 | 13.59 | 3850 |
| Lamiaceae | *Nepeta glutinosa* | 17 | M | sexual | 1.58 | 7.97 | 3880 |
| Lamiaceae | *Nepeta leucolaena* | 10 | O | sexual | 1.53 | 13.10 | 3896 |
| Lamiaceae | *Nepeta longibracteata* | 15 | M | sexual | 1.52 | 10.33 | 5316 |
| Lamiaceae | *Stachys tibetica* | 10 | O | sexual | 1.47 | 58.05 | 3518 |
| Lamiaceae | *Thymus linearis* | 10 | O | sexual | 1.49 | 9.49 | 4293 |
| Morinaceae | *Morina coulteriana* | 3 | O | sexual | 1.51 | 71.86 | 3402 |
| Onagraceae | *Epilobium latifolium* | 14 | M | × | × | × | 4548 |
| Orobanchaceae | *Euphrasia foliosa* | 10 | O | sexual | 1.42 | 55.57 | 4060 |
| Orobanchaceae | *Leptorhabdos parviflora* | 6 | O | sexual | 1.44 | 26.31 | 2984 |
| Orobanchaceae | *Pedicularis bicornuta* | 9 | O | sexual | 1.48 | 43.27 | 4102 |
| Orobanchaceae | *Pedicularis cheilanthifolia* | 16 | O | sexual | 1.47 | 26.89 | 4871 |
| Papaveraceae | *Meconopsis aculeata* | 25 | O | sexual | 1.57 | 95.06 | 4433 |
| Plantaginaceae | *Picrorhiza kurroa* | 21 | O | sexual | 1.49 | 75.33 | 3910 |
| Plantaginaceae | *Plantago depressa* | 10 | O | sexual | 1.51 | 20.71 | 3184 |
| Plantaginaceae | *Plantago himalaica* | 10 | O | sexual | 1.52 | 27.00 | 4182 |
| Plantaginaceae | *Veronica biloba* | 16 | O | sexual | 1.52 | 62.61 | 3985 |
| Plumbaginaceae | *Acantholimon lycopodioides* | 6 | O | × | × | × | 4183 |
| Poaceae | *Alopecurus himalaicus* | 10 | M | sexual | 1.46 | 45.25 | 4111 |
| Poaceae | *Bromus oxyodon* | 3 | M | sexual | 1.46 | 5.02 | 3386 |
| Poaceae | *Bromus tectorum* | 4 | M | sexual | 1.48 | 15.47 | 3202 |
| Poaceae | *Calamagrostis stoliczkae* | 12 | O | sexual | 1.46 | 41.17 | 4116 |
| Poaceae | *Catabrosa aquatica* | 3 | M | sexual | 1.46 | 52.66 | 4177 |
| Poaceae | *Digitaria stewartiana* | 3 | M | sexual | 1.46 | 10.93 | 3040 |

| | | | | | | | |
|---|---|---|---|---|---|---|---|
| Poaceae | *Echinochloa crus-galli* | 6 | M | sexual | 1.46 | 11.77 | 2910 |
| Poaceae | *Elymus cognatus* | 10 | O | sexual | 1.44 | 35.09 | 3808 |
| Poaceae | *Elymus dentatus* | 3 | M | sexual | 1.57 | 71.28 | 3821 |
| Poaceae | *Elymus jacquemontii* | 10 | O | sexual | 1.49 | 26.81 | 4992 |
| Poaceae | *Elymus schrenkianus* | 10 | M | sexual | 1.50 | 46.92 | 4977 |
| Poaceae | *Elymus schugnanicus* | 3 | M | sexual | 1.61 | 38.87 | 4424 |
| Poaceae | *Enneapogon persicus* | 3 | M | × | × | × | 2809 |
| Poaceae | *Eragrostis minor* | 6 | M | sexual | 1.42 | 19.00 | 2964 |
| Poaceae | *Eremopoa altaica* | 3 | M | sexual | 1.48 | 32.65 | 3830 |
| Poaceae | *Festuca coelestis* | 6 | M | sexual | 1.46 | 67.77 | 4901 |
| Poaceae | *Festuca nitidula* | 9 | M | sexual | 1.56 | 43.13 | 4663 |
| Poaceae | *Festuca non-coelestis* | 6 | M | sexual | 1.57 | 49.91 | 5412 |
| Poaceae | *Festuca olgae* | 21 | M | apom./sex | 1.48, 2.85 | 48.14 | 4773 |
| Poaceae | *Festuca wallichiana* | 6 | M | sexual | 1.58 | 78.56 | 4055 |
| Poaceae | *Hordeum brevisubulatum* | 13 | O | sexual | 1.45 | 27.29 | 4599 |
| Poaceae | *Leymus secalinus* | 8 | O | sexual | 1.52 | 58.44 | 4663 |
| Poaceae | *Melica persica* | 6 | M | × | × | × | 3996 |
| Poaceae | *Phalaris canariensis* | 6 | M | sexual | 1.42 | 55.84 | 3220 |
| Poaceae | *Phleum alpinum* | 12 | M/O | sexual | 1.48 | 19.56 | 3524 |
| Poaceae | *Poa alpina* | 8 | M | apomictic | 2.64 | 37.00 | 3896 |
| Poaceae | *Poa attenuata* | 26 | M | apomictic | 1.92, 2.90 | 23.48 | 5444 |
| Poaceae | *Poa pratensis subsp. pratensis* | 7 | M | apomictic | 2.72 | 37.55 | 4938 |
| Poaceae | *Poa pratensis subsp. staintonii* | 7 | M | apom./sex | 1.47, 2.44 | 33.47 | 4938 |
| Poaceae | *Poa sterilis* | 10 | M | apom./sex | 1.48, 2.37 | 19.69 | 3559 |
| Poaceae | *Poa suruana* | 7 | M | sexual | 1.50 | 56.46 | 4276 |
| Poaceae | *Puccinellia ladakhensis* | 6 | M | sexual | 1.50 | 30.67 | 4678 |
| Poaceae | *Puccinellia pauciramea* | 6 | M | sexual | 1.49 | 45.40 | 4550 |
| Poaceae | *Puccinellia stapfiana* | 6 | M | sexual | 1.52 | 51.60 | 4590 |
| Poaceae | *Stipa capillata* | 2 | M | × | × | × | 3447 |
| Poaceae | *Stipa koelzii* | 6 | M | sexual | 1.57 | 40.34 | 1485 |
| Poaceae | *Stipa mongholica* | 6 | M | sexual | 1.53 | 51.37 | 4375 |
| Poaceae | *Stipa orientalis* | 7 | M | sexual | 1.51 | 30.32 | 3911 |
| Poaceae | *Stipa splendens* | 20 | M | apomictic | 2.14, 2.64 | 30.42 | 3293 |
| Poaceae | *Stipa subsessiliflora* | 16 | M | sexual | 1.48 | 45.59 | 4861 |
| Poaceae | *Trikeraia oreophila* | 6 | M | sexual | 1.50 | 16.60 | 4859 |
| Poaceae | *Trisetum spicatum* | 40 | M | sexual | 1.51 | 53.06 | 5301 |
| Polygonaceae | *Bistorta affinis* | 6 | O | sexual | 1.35 | 61.13 | 4303 |
| Polygonaceae | *Knorringia pamirica* | 13 | M | sexual | 1.50 | 18.67 | 4710 |
| Polygonaceae | *Oxyria digyna* | 18 | O | sexual | 1.50 | 66.91 | 3531 |
| Polygonaceae | *Polygonum cognatum* | 10 | M | sexual | 1.48 | 15.19 | 4767 |
| Polygonaceae | *Polygonum rumicifolium* | 13 | O | sexual | 1.42 | 44.84 | 3861 |
| Polygonaceae | *Rheum tibeticum* | 3 | O | sexual | 1.47 | 28.62 | 4249 |
| Polygonaceae | *Rheum webbianum* | 3 | O | sexual | 1.49 | 23.16 | 4020 |
| Polygonaceae | *Rumex patientia* | 9 | M | × | × | × | 3271 |
| Primulaceae | *Primula macrophylla* | 10 | O | sexual | 1.47 | 66.34 | 5360 |
| Primulaceae | *Primula moorcroftiana* | 9 | O | sexual | 1.47 | 73.28 | 5360 |

| | | | | | | | |
|---|---|---|---|---|---|---|---|
| Ranunculaceae | *Anemone rupicola* | 10 | O | sexual | 1.53 | 76.52 | 3977 |
| Ranunculaceae | *Aquilegia fragrans* | 9 | M | sexual | 1.47 | 96.29 | 3309 |
| Ranunculaceae | *Aquilegia moorcroftiana* | 16 | M/O | sexual | 1.48 | 95.35 | 4040 |
| Ranunculaceae | *Batrachium flavescens* | 9 | M | sexual | 1.49 | 84.76 | 4150 |
| Ranunculaceae | *Clematis tangutica* | 9 | O | sexual | 1.50 | 66.23 | 4202 |
| Ranunculaceae | *Clematis sp.* | 6 | O | sexual | 1.53 | 66.50 | NA |
| Ranunculaceae | *Clematis sp.* | 6 | O | × | × | × | NA |
| Ranunculaceae | *Delphinium brunonianum* | 17 | M | sexual | 1.50 | 92.17 | 5266 |
| Ranunculaceae | *Halerpestes lancifolia* | 9 | M | apomictic | 3.18 | 22.64 | 4014 |
| Ranunculaceae | *Pulsatilla wallichiana* | 9 | O | sexual | 1.51 | 78.45 | 4180 |
| Ranunculaceae | *Ranunculus lobatus* | 9 | O | sexual | 1.56 | 35.62 | 5272 |
| Ranunculaceae | *Ranunculus membranaceus* | 9 | O | apomictic | 3.00 | 9.04 | 3500 |
| Ranunculaceae | *Thalictrum foetidum* | 9 | O | sexual | 1.65 | 78.90 | 3483 |
| Rhamnaceae | *Rhamnus prostrata* | 6 | M | sexual | 1.50 | 32.34 | 3911 |
| Rosaceae | *Comarum salesovianum* | 24 | M/O | sexual | 1.58 | 11.65 | 3901 |
| Rosaceae | *Chamaerhodos sabulosa* | 11 | O | × | × | × | 4980 |
| Rosaceae | *Potentilla atrosanguinea* | 10 | M | sexual | 1.54 | 6.78 | 3708 |
| Rosaceae | *Potentilla bifurca* | 14 | M/O | sexual | 1.54 | 7.56 | 4912 |
| Rosaceae | *Potentilla evestita* | 12 | O | × | × | × | 4650 |
| Rosaceae | *Potentilla gelida* | 16 | O | × | × | × | 5260 |
| Rosaceae | *Potentilla multifida* | 9 | O | sexual | 1.50 | 8.94 | 4647 |
| Rosaceae | *Potentilla pamirica* | 30 | M | apomictic | 3.06 | 4.86 | 5466 |
| Rosaceae | *Potentilla sericea* | 13 | M | apomictic | 3.04 | 2.40 | 4980 |
| Rosaceae | *Potentilla sojakii* | 46 | M | apomictic | 3.06 | 2.90 | 4517 |
| Rosaceae | *Potentilla turczaninowiana* | 16 | M | sexual | 1.59 | 9.51 | 4615 |
| Rosaceae | *Potentilla venusta* | 38 | M | × | × | × | 4785 |
| Rosaceae | *Rosa webbiana* | 9 | O | × | × | × | 3685 |
| Rosaceae | *Sibbaldia cuneata* | 7 | O | × | × | × | 4181 |
| Rosaceae | *Sorbus tianschanica* | 2 | O | sexual | 1.52 | 24.92 | 3271 |
| Rubiaceae | *Galium pauciflorum* | 9 | O | sexual | 1.47 | 26.22 | 3634 |
| Rubiaceae | *Rubia tibetica* | 10 | O | sexual | 1.51 | 55.52 | 3963 |
| Salicaceae | *Salix flabellaris* | 9 | M | × | × | × | 4113 |
| Saxifragaceae | *Saxifraga hirculoides* | 25 | M | sexual | 1.52 | 78.69 | 5066 |
| Saxifragaceae | *Saxifraga hirculus* | 8 | O | sexual | 1.50 | 84.97 | 5066 |
| Saxifragaceae | *Saxifraga nanella* | 24 | M | sexual | 1.47 | 67.63 | 5569 |
| Scrophulariaceae | *Scrophularia dentata* | 71 | O | sexual | 1.47 | 42.09 | 4586 |
| Solanaceae | *Physochlaina praealta* | 16 | M/O | sexual | 1.50 | 42.94 | 3760 |
| Tamaricaceae | *Myricaria germanica* | 15 | M | × | × | × | 4334 |
| Urticaceae | *Urtica hyperborea* | 25 | O | sexual | 1.48 | 63.42 | 5053 |
| Valerianaceae | *Valeriana himalayana* | 10 | O | sexual | 1.49 | 9.50 | 4097 |

**Supplementary Table S5**: Summary of percentage of endosperm occurring in individual families. Percentage of endosperm in a seed was calculated as the ratio of count of nuclei of the endosperm peak to the sum of both peaks. Blue color indicate values less than 50 %, red color indicate values greater than 50 %. SD was calculated for each family.

| **family** | **% of endosperm** | **SD** |
|---|---|---|
| Alliaceae | 50.98 | 27.22 |
| Amaranthaceae | 1.47 | 0.09 |
| Apiaceae | 76.14 | 15.90 |
| Apocynaceae | 41.63 | 7.09 |
| Asteraceae | 14.63 | 8.61 |
| Balsamiaceae | 7.89 | 5.33 |
| Biebersteiniaceae | 23.75 | 8.05 |
| Boraginaceae | 12.03 | 4.49 |
| Brassicaceae | 14.08 | 14.67 |
| Capparaceae | 21.43 | 2.60 |
| Caprifoliaceae | 80.47 | 6.62 |
| Caryophyllaceae | 10.29 | 5.45 |
| Cuscutaceae | 24.06 | 0.98 |
| Cyperaceae | 52.46 | 7.41 |
| Euphorbiaceae | 60.21 | 9.33 |
| Fabaceae | 7.24 | 3.27 |
| Fumariaceae | 61.99 | 19.99 |
| Gentianaceae | 80.42 | 4.68 |
| Grossulariaceae | 85.27 | 0.72 |
| Chenopodiaceae | 3.27 | 1.08 |
| Iridaceae | 82.96 | 0.00 |
| Juncaceae | 33.92 | 6.02 |
| Lamiaceae | 15.64 | 14.72 |
| Morinaceae | 71.86 | 0.00 |
| Orobanchaceae | 39.13 | 13.94 |
| Papaveraceae | 95.06 | 2.62 |
| Plantaginaceae | 50.55 | 24.98 |
| Poaceae | 39.00 | 16.88 |
| Polygonaceae | 31.48 | 16.43 |
| Primulaceae | 69.81 | 6.73 |
| Ranunculaceae | 65.44 | 28.98 |
| Rhamnaceae | 32.34 | 0.87 |
| Rosaceae | 8.35 | 4.86 |
| Rubiaceae | 40.87 | 14.98 |
| Saxifragaceae | 78.94 | 8.88 |
| Scrophulariaceae | 42.09 | 10.91 |
| Solanaceae | 42.94 | 1.98 |
| Urticaceae | 57.58 | 12.92 |
| Valerianaceae | 9.50 | 1.56 |
